# Analysis of Liquid Ensembles for Enhancing the Performance and Accuracy of Liquid State Machines

**DOI:** 10.3389/fnins.2019.00504

**Published:** 2019-05-28

**Authors:** Parami Wijesinghe, Gopalakrishnan Srinivasan, Priyadarshini Panda, Kaushik Roy

**Affiliations:** School of Electrical and Computer Engineering, Purdue University, West Lafayette, IN, United States

**Keywords:** liquid state machines, ensembles, spiking neural networks, separation property, approximation property, discriminant ratio

## Abstract

Liquid state machine (LSM), a bio-inspired computing model consisting of the input sparsely connected to a randomly interlinked reservoir (or liquid) of spiking neurons followed by a readout layer, finds utility in a range of applications varying from robot control and sequence generation to action, speech, and image recognition. LSMs stand out among other Recurrent Neural Network (RNN) architectures due to their simplistic structure and lower training complexity. Plethora of recent efforts have been focused toward mimicking certain characteristics of biological systems to enhance the performance of modern artificial neural networks. It has been shown that biological neurons are more likely to be connected to other neurons in the close proximity, and tend to be disconnected as the neurons are spatially far apart. Inspired by this, we propose a group of locally connected neuron reservoirs, or an ensemble of liquids approach, for LSMs. We analyze how the segmentation of a single large liquid to create an ensemble of multiple smaller liquids affects the latency and accuracy of an LSM. In our analysis, we quantify the ability of the proposed ensemble approach to provide an improved representation of the input using the Separation Property (SP) and Approximation Property (AP). Our results illustrate that the ensemble approach enhances class discrimination (quantified as the ratio between the SP and AP), leading to better accuracy in speech and image recognition tasks, when compared to a single large liquid. Furthermore, we obtain performance benefits in terms of improved inference time and reduced memory requirements, due to lowered number of connections and the freedom to parallelize the liquid evaluation process.

## 1. Introduction

Over the past few decades, artificial neural algorithms have developed to an extent that they can perform more human-like functions. Recurrent Neural Networks (RNNs) and their variants such as Long Short Term Memory (LSTM) networks have become the state-of-the-art for processing spatio-temporal data. The massive RNNs of today, can describe images in natural language (Xie, 2017), produce handwriting (Graves, 2013), and even make phone calls to book appointments (Yaniv and Yossi, 2018). Such fascinating, human-like capabilities are obtained at the cost of increased structural and training complexity, and thus significant power consumption, storage requirements, and delay.

In this work we focus on a particular type of spiking RNN; the Liquid State Machine (LSM) (Maass et al., 2002). An LSM consists of a set of inputs sparsely connected to a randomly and recurrently interlinked pool of spiking neurons called the “liquid”. The liquid is connected to an output classifier, which can be trained using standard methods such as Spike Timing Dependent Plasticity (STDP), backpropagation, delta rule *etc*. (Kötter, 2012) and using enhanced learning rules (Roy et al., 2013). LSMs have been used for a variety of applications including robot control (Urbain et al., 2017), sequence generation (Panda and Roy, 2017), decoding actual brain activity (Nikolić et al., 2009), action recognition (Panda and Srinivasa, 2018), speech recognition (Maass et al., 2002; Verstraeten et al., 2005; Goodman and Ventura, 2006; Zhang et al., 2015; Wu et al., 2018; Zhang and Li, 2019), and image recognition (Grzyb et al., 2009; Wang and Li, 2016; Srinivasan et al., 2018; Zhang and Li, 2019).

LSMs gained their popularity due to two main reasons. First, the LSM architecture is neuro-inspired. By conserving energy via spike-based operation, the brain has evolved to achieve its prodigious signal-processing capabilities using orders of magnitude less energy than the state-of-the-art supercomputers (Anastassiou et al., 2011; Cruz-Albrecht et al., 2012). Therefore, with the intention to pave pathways to low power neuromorphic computing, much consideration is given to realistic artificial brain modeling (Waldrop, 2012; Neftci et al., 2016; Wijesinghe et al., 2018). Furthermore, the gene regulation network (GRN) of the Bacterium “Escherichia Coli” (E-Coli) was experimentally assessed and shown to behave similar to an LSM (Jones et al., 2007). The E. Coli has the capacity for perceptual categorization, especially for discrimination between complex temporal patterns of chemical inputs. Second, LSMs have simple structure and lower training complexity among other RNNs. The claim is that, sufficiently large and complex liquids inherently possess large computational power for real-time computing. Therefore, it is not necessary to “construct” circuits to achieve substantial computational power. However, such simple structure of LSMs comes with an accuracy trade-off. A plethora of work in the literature suggests mechanisms for improving the accuracy of LSMs including training the liquid connections (Wang and Li, 2016) and involving multiple layers of liquids to form deep LSMs (Xue et al., 2016). Despite the accuracy improvement, these mechanisms found in literature tend to alter the standard simple structure of LSMs. Choosing an LSM for a particular application and improving its accuracy at the cost of added complexity, nonetheless questions the motivation behind choosing an LSM in the first place.

Without deviating from the inherent simplicity of the LSM structure, several basic approaches can be used to improve its accuracy. One such fundamental approach is to increase the number of neurons within the liquid. However, the number of connections within the liquid also increases following a quadratic relationship with the number of neurons. Furthermore, the sensitivity of accuracy to the liquid neuron count decreases with the number of neurons beyond a certain point. In other words, enlarging the liquid introduces scalability challenges, and the accompanied cost tends to veil the accuracy benefits. The percentage connectivity also plays a role in improving the accuracy. Either high or low percentage connectivity results in accuracy degradation, signaling the existence of an optimum connectivity.

Note, there are two key properties that measure the capacity of an LSM: *separation* and *approximation* (Maass et al., 2002). Aforementioned basic approaches; changing the number of neurons and connectivity in the liquid, indeed has an impact on the above measures. Based on separation and approximation, we propose an “ensemble of liquids approach” that can improve the classification accuracy (compared to a single large LSM) with reduced connectivity. The approach is scalable and preserves the simplicity of the network structure. In our ensemble of liquids approach, we split a large liquid into multiple smaller liquids. These resultant liquids can be evaluated in parallel since they are independent of each other, which leads to performance benefits. Furthermore, for a given percentage connectivity, the number of connections available in the ensemble approach is less than that of a single liquid with the same number of neurons. This reduces the storage requirement of the LSM as well. We used a variant of the Fisher's linear discriminant ratio (Fisher, 1936; Fukunaga and Mantock, 1983) (the ratio between the separation and approximation) to quantify how well the ensemble of liquids represents the spatio-temporal input data. We observed that increasing the liquid count beyond a certain point reduces the accuracy of the LSM. This signals the existence of an optimum number of liquids, which is highly dependent upon the application and the number of neurons in the liquid. We show that dividing the liquid provides both accuracy and performance benefits for spatial and temporal pattern recognition tasks, on standard speech and image data sets.

The “ensemble” concept has been previously used (Yao et al., 2013) for echo state networks or ESNs (Jaeger, 2007), which are similar in architecture to LSMs but use artificial rate-based neurons. Rather than using a single ESN predictor, multiple predictors (component predictors) were used and their predictions were combined together to obtain the final outcome. This approach was proposed to avoid the instability of the output of each individual predictor, since the input and internal connection weights are assigned randomly. The final ensemble outcome was obtained by averaging the predictions of the component predictors. The approach in Yao et al. (2013) is different from our work since we design the ensemble of liquids by removing certain connections from a bigger reservoir. Furthermore, only a single classifier is used at the output in our work in contrast to Yao et al. (2013). The authors in Maass et al. (2002) conducted a small experiment with two time-varying signals, which shows that using four liquids is better than using a single liquid in terms of enhancing the separation property. However, in their experiments, the four liquids in total have four times the number of neurons as the single liquid case. Therefore, it is not obvious whether the improvement in separation is solely due to having four “separate” liquids. The increased number of neurons itself might have played a role in enhancing the separation. In contrast, we analyze the effects of dividing a large liquid into multiple smaller units, while leaving the total number of neurons the same. Research (Srinivasan et al., 2018) also shows that multiple liquids perform better than a single liquid, at higher number of neurons. The input to liquid connections in Srinivasan et al. (2018) were trained in an unsupervised manner. Also note that each liquid was fed with distinct parts of an input, and hence is different from this work.

## 2. Materials and Methods

### 2.1. Liquid State Machine (baseline)

In this section, we explain the structure and training of the LSM used in this work. The conventional structure of an LSM consists of an input layer sparsely connected to a randomly interlinked pool of spiking neurons called the liquid. The liquid is then connected to a classifier which has synaptic weights that could be learnt using supervised training algorithms for inference.

#### 2.1.1. Liquid Neurons

**Figure 1:**
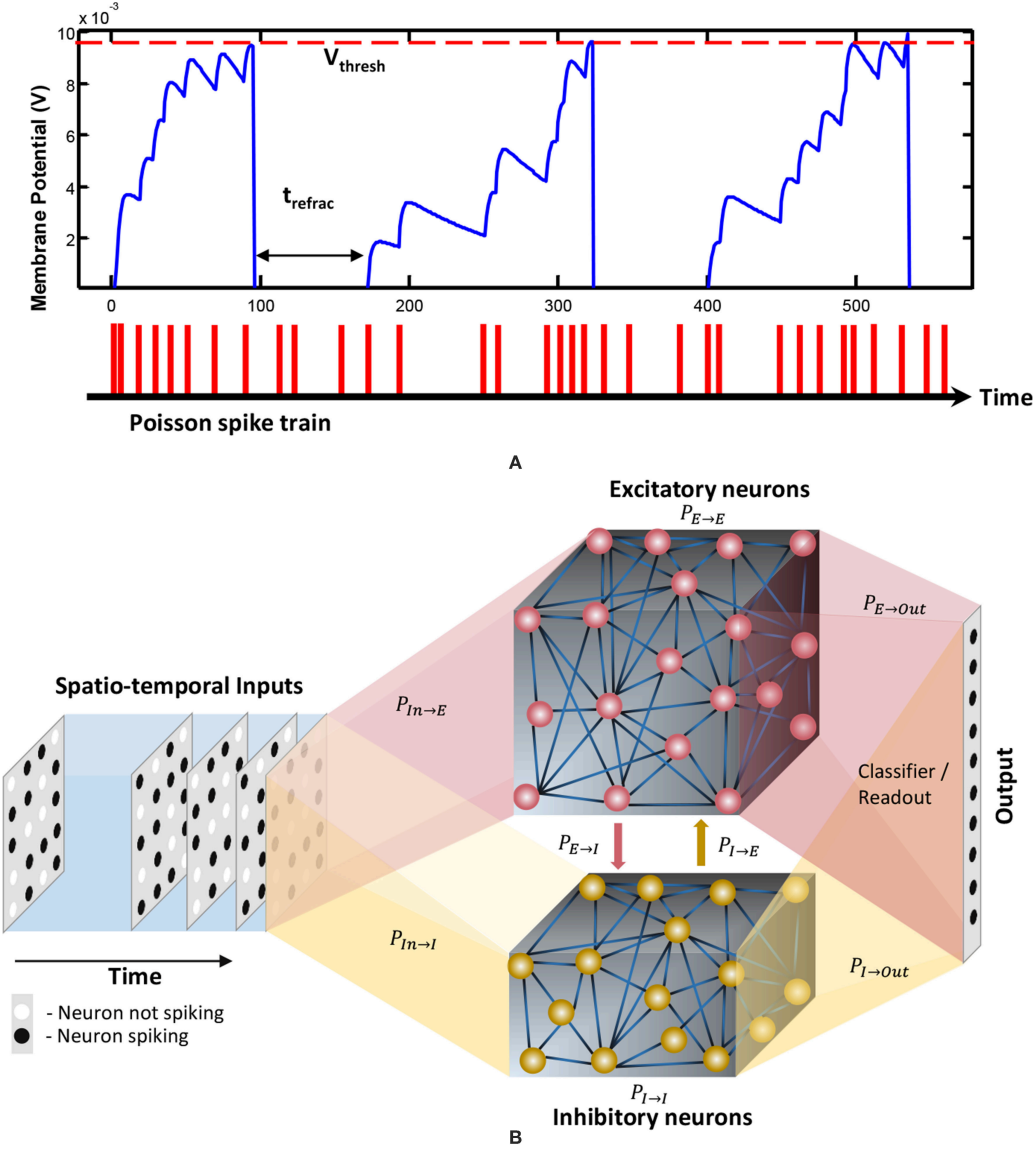
**(A)** The dynamics of the membrane potential (*V*) of a spiking neuron. Each spike shown below the graph will increase the membrane potential. When *V* reaches the threshold *V*_*thresh*_, the neuron will generate a spike and *V* will drop to the rest potential *V*_*rest*_ for a *t*_*refrac*_ duration of time. This duration is called the refractory period, and the neuron stays idle within this period. Reprinted with the permission from Liyanagedera et al. (2017). **(B)** The structure of the liquid state machine. The input is connected to a reservoir with two types of neurons; inhibitory and excitatory. The reservoir is then connected to a classifier which is typically trained using supervised learning methods. The percentage connectivity between different types of *pre* and *post* neurons (*P*_*pre*→*post*_) are as indicated in the figure.

The neurons within the liquid are leaky integrate-and-fire neurons (Abbott, 1999) of two types; excitatory and inhibitory neurons. The number of excitatory (*E*) neurons and inhibitory (*I*) neurons were selected according to a 4:1 ratio, as observed in the auditory cortex (Wehr and Zador, 2003). The membrane potential (*V*) of a neuron increases/decreases as a pre excitatory/inhibitory neuron connected to it spikes that is described by

(1) $$\begin{array}{r} \tau \frac{dV}{dt}=\left(E_{rest}-V\right)+g_{e}\left(E_{exc}-V\right)+g_{i}\left(E_{inh}-V\right) \end{array}$$

where *E*_*rest*_ is the resting membrane potential, τ is the membrane potential decay time constant, *E*_*exc*_ and *E*_*inh*_ are the equilibrium potentials of excitatory and inhibitory synapses, and *g*_*e*_ and *g*_*i*_ are the conductance values of the excitatory and inhibitory synapses, respectively. As the membrane potential reaches a certain threshold, the neuron generates a spike. The membrane potential drops to its reset potential upon generating a spike as shown in Figure 1A, and then enters its refractory period *t*_*refrac*_ during which it produces no further spikes. The formulations elaborated in Diehl and Cook (2015) were used for modeling the dynamics of the spiking neurons.

#### 2.1.2. Liquid Connections

The input is sparsely connected to the liquid (*In*→*E* connections). The percentage input to liquid connectivity (*P*_*IN*→*E*_) plays an important role in achieving good accuracy as will be explained in section 3.4.2. The liquid is composed of connections from excitatory to excitatory neurons (*E*→*E*), excitatory to inhibitory neurons (*E*→*I*), inhibitory to excitatory neurons (*I*→*E*), and inhibitory to inhibitory neurons (*I*→*I*). In our notation, the first letter indicates the pre-neuron type (*PRE*) and the second letter denotes the post-neuron type (*POST*). The selected percentage connectivity (*P*_*IN*→*E*_ , *P*_*E*→*E*_ , *P*_*E*→*I*_ , *P*_*I*→*E*_, *P*_*I*→*I*_) within the liquid are shown in Table 1. These percentage connectivity values worked the best in terms of accuracy, for the neuron parameter selections in this work shown in Table 2. The strengths of all the connections ($W\in {\left[0,1\right]}^{N_{PRE}\times N_{POST}}$) were selected randomly (Maass et al., 2002) from a uniform distribution *U*(0, 1) (Toledo-Suárez et al., 2014; Srinivasan et al., 2018). A randomly generated mask ($M\in {\left\{0,1\right\}}^{N_{PRE}\times N_{POST}},m_{ij}\in M$) decides which connections exist to obtain the desired sparsity/percentage connectivity ($P_{PRE\to POST}=\frac{\underset{\left(\forall i,\forall j\right)}{\sum }m_{ij}}{\left(N_{PRE}\times N_{POST}\right)}\times 100\%$). Here *N*_*PRE*_ and *N*_*POST*_ are the number of *PRE* and *POST* neurons, respectively. The dynamic conductance change model was used for synapses. i.e., when a pre-synaptic neuron fires, the synaptic conductance instantaneously changes according to their strengths and then decays exponentially with a time constant (Diehl and Cook, 2015). Following equation shows the dynamics of a synapse (*g*_*e*_) with an excitatory pre-neuron. τ_*g*_*e*__ is the decay time constant. This is similar to the post-synaptic current model in Maass et al. (2003).

(2) $$\begin{array}{r} \tau_{g_{e}}\frac{dg_{e}}{dt}=-g_{e} \end{array}$$

**Table 1:** Percentage connectivity within the liquid.

**Type of connectivity**	**Percentage connectivity (Speech recognition)**		**Percentage connectivity (Image recognition)**	
	**TI-alpha(%)**	***TI*−10(%)**	**MNIST(%)**	**E-MNIST(%)**
Input–Excitatory	34	23	10	10
Input–Inhibitory	0	0	0	0
Excitatory–Excitatory	40	40	40	40
Excitatory–Inhibitory	40	40	40	40
Inhibitory–Excitatory	50	50	50	50
Inhibitory–Inhibitory	0	0	0	0

**Table 2:** Spiking neuron parameters of the liquid state machine.

**Parameter name**	**Parameter value**
Excitatory weight decay time constant, *t*_*ge*_	1 ms
Inhibitory weight decay time constant, *t*_*gi*_	2 ms
Threshold inhibitory, *thresh*_*i*_	−40 mV
Threshold excitatory, *thresh*_*e*_	−52 mV
Inhibitory rest potential, *v*_*rest, i*_	−60 mV
Excitatory rest potential, *v*_*rest, e*_	−65 mV

#### 2.1.3. Output Classifier

The liquid is connected to a classifier which is trained in a supervised manner. As suggested in Maass et al. (2002), a memory-less readout (the readout is not required to retain any memory of previous states) can be used to classify the states of the liquid. The liquid state in this work is the normalized spike count of the excitatory neurons (Kaiser et al., 2017) within a duration of *T*, when the input is applied. There is a liquid state vector ($s_{i}\in {\left[0,1\right]}^{N_{E}}$, *N*_*E*_ is the number of excitatory neurons) per applied input (*i*). The collection of all the state vectors were then used to train the classifier using gradient descent error backpropagation algorithm (Rumelhart et al., 1986), similar to Srinivasan et al. (2018). By doing this, we do discard some temporal information. However, since we do not use “anytime-speech-recognition” (a liquid with a classifier which is capable of recognizing a speech input, before the entire temporal signal is applied to the liquid) proposed in Maass et al. (2004), the above classification method is sufficient to achieve reasonable accuracy (as per the accuracy values reported in other LSM works) for the applications we are considering in this work.

### 2.2. Ensemble Approach for LSMs

**Figure 2:**
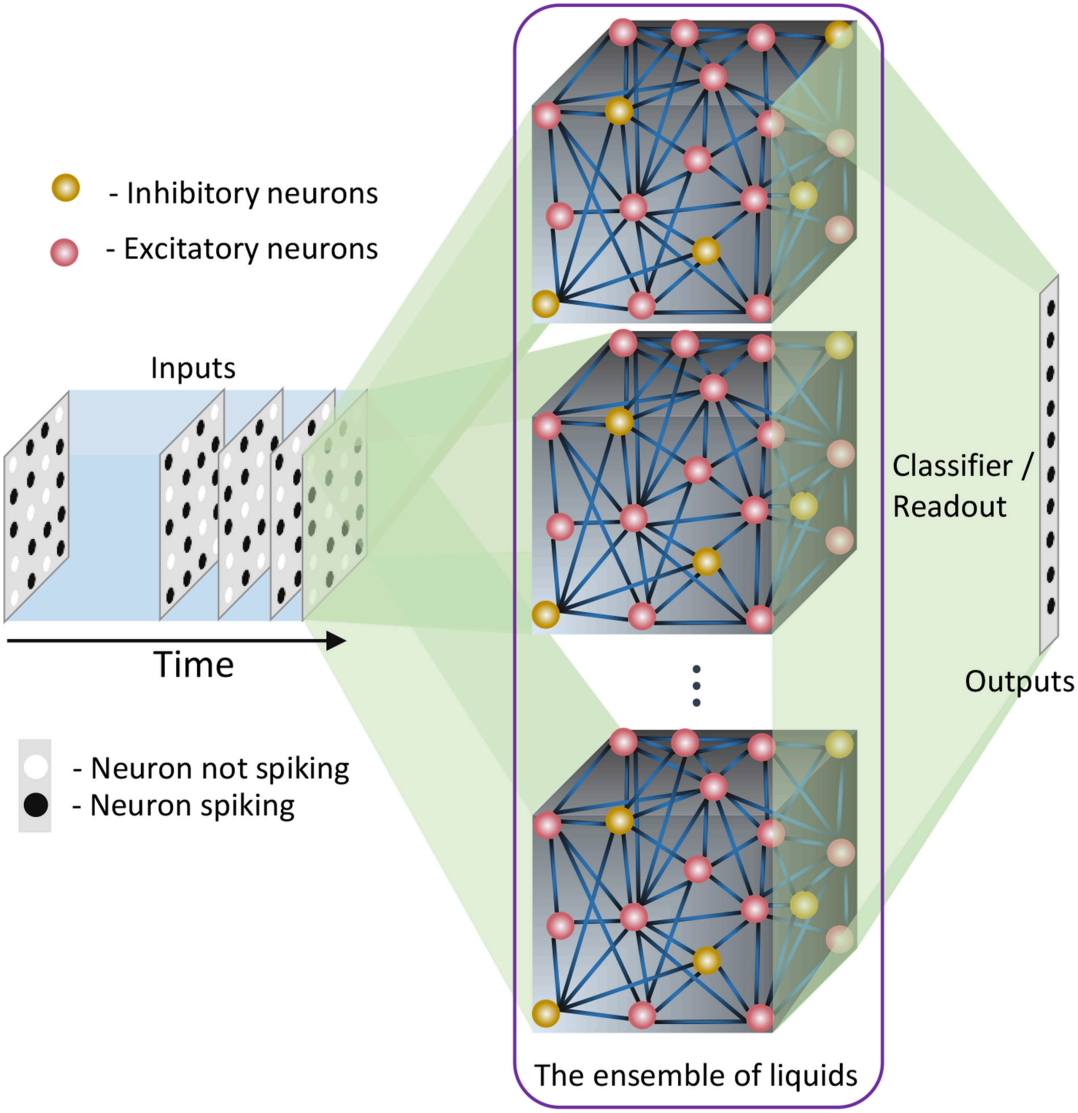
The structure of the ensemble approach. The liquid in the standard LSM is split up to create an ensemble of smaller liquids. The input is sparsely connected to all the liquids. The output of all the liquids are concatenated to form one large liquid state vector, and connected to a single readout that is trained using supervised learning methods.

In this section, we explain our proposed ensemble of liquids approach, which improves the scalability of LSMs. The proposed approach is different from the ensemble works available in literature on a variety of network types (feed-forward fully connected spiking and analog neural networks), where multiple classified outputs of independently trained networks are combined together to increase the probability of correct classification (Jacobs et al., 1991; Shim et al., 2016). In this work, we analyze the impact of dividing a reservoir, such that all the resultant small reservoirs can potentially be evaluated in parallel, for an applied input. As explained in the previous section, the typical structure of an LSM has an input, a liquid where neurons are sparsely interlinked, and a readout trained using supervised learning methods (Figure 1B). In our ensemble approach, the same number of liquid neurons (*N*_*tot*_) is divided to create an *N*_*ens*_ number of smaller liquids, as shown in Figure 2. While dividing the liquid, the number of excitatory neurons ($N_{E}^{i}$) to inhibitory neurons ($N_{I}^{i}$) ratio in the *i*th (*i* = 0, 1, …, *N*_*ens*_) liquid is maintained at 4:1. The percentage connectivity is also adjusted to suit the new reduced number of neurons ($\frac{N_{tot}}{N_{ens}}$) in a liquid. This is done by first creating a standard LSM explained in the previous section with $\frac{N_{tot}}{N_{ens}}$ number of neurons and adjusting all the percentage connectivity values till we get a reasonable accuracy. Then the input to liquid percentage connectivity (*P*_*IN*→*E*_) was exhaustively changed until the accuracy peaks for a given application, which is then used for all the experiments reported in this work.

Each small liquid has its own liquid state vector, which is the normalized spike count of all the excitatory neurons in the respective liquid within a duration of *T*, as explained in the previous section. All the state vectors produced by each individual liquid in the ensemble are concatenated to form one large state vector per input. Note that the length of the concatenated state vectors are the same for both the single liquid case (baseline) and for the ensemble of liquids, since the total number of neurons are held constant for a fair comparison. The concatenated state vector is used to train a single readout using gradient descent backpropagation. This division of one large liquid to form an ensemble of liquids enhances class discrimination associated with LSMs as elaborated in the next section.

### 2.3. Properties of LSMs

Two macroscopic properties of an LSM, namely, Separation Property (SP) and Approximation Property (AP), can be used to quantify a liquid's ability to provide an improved projection of the input data. With respect to classification applications, SP gives a measure of the liquid's ability to separate input instances that belong to different classes. AP, on the other hand, gives a measure of the closeness of the liquid's representation of two inputs that belong to the same class.

Several methods of quantifying the SP and AP as a measure of the computational power (kernel quality) of an LSM are suggested in Maass et al. (2002, 2005). Two methods of measuring the SP are *pairwise separation property* and *linear separation property*. The pairwise separation property is the distance between two continuous time states of the liquid (*x*_*u*_(*t*) and *x*_*v*_(*t*)), resulting from two different input spike trains (*u*(*t*) and *v*(*t*)). Here the continuous time states *x*(*t*) are defined as the vector of output values of linear filters with exponential decay (with time constant 30*ms* Maass et al., 2002) at time *t*. The distance can be calculated by the Euclidean norm between *x*_*u*_(*t*_*n*_) and *x*_*v*_(*t*_*n*_) at sample point *t*_*n*_. The final pairwise separation property can be evaluated by obtaining the average across all the sampled instances (at *t*_*n*_), as explained in the following equation

(3) $$\begin{array}{r} SP_{pw}=\frac{1}{N_{samples}}\overset{N_{samples}}{\underset{n=1\left(0<t_{n}<T\right)}{\sum }}||x_{u}\left(t_{n}\right)-x_{v}\left(t_{n}\right)|| \end{array}$$

where *N*_*samples*_ is the number of sample points. The pairwise separation property (*SP*_*pw*_) can be used as a measure of the kernel quality for two given inputs. However, most real life applications deal with more than two input spike trains. To address this, linear separation property is proposed as a more suitable quantitative measure to evaluate the computational power of a liquid in an LSM. The linear separation property (*SP*_*lin*_) is the rank of the *N* × *m* matrix *M*_*s*_, which contains the continuous time states (*x*_*u*_1__(*t*_0_), …, *x*_*u*_*m*__(*t*_0_)) of the liquid as its columns. The continuous time state *x*_*u*_*i*__(*t*_0_) is the liquid response to the input *u*_*i*_ (these inputs are from the training set), sampled at *t* = *t*_0_. If the rank of the matrix is *m*, it guarantees that any given assignment of target outputs *y*_*i*_ ∈ ℝ at time *t*_0_ can be attained by means of a linear readout (Maass et al., 2005). The rank of *M*_*s*_ is the degree of freedom the linear readout has, when mapping *x*_*u*_*i*__ to *y*_*i*_. Even though the rank is < *m*, it can still be used as a measure of kernel quality of the LSM (Maass et al., 2005).

(4) $$\begin{array}{l} M_{s}=[x_{u_{1}}(t_{0}),...,x_{u_{i}}(t_{0}),...,x_{u_{m}}(t_{0})]\\ \;SP_{lin}=rank(M_{s}) \end{array}$$

The AP of the LSM can also be measured by the aforementioned rank concept as shown in Maass et al. (2005); Roy and Basu (2016). Instead of using significantly different examples in the training set, now the continuous time states $x_{u_{i}^{j}}\left(t_{0}\right)$ of the liquid are measured by feeding jittered versions of *u*_*i*_ ($u_{i}^{j}$) to the liquid. The rank of the matrix *M*_*a*_ that has *m* such continuous time states $x_{u_{1}^{j}}\left(t_{0}\right),\ldots ,x_{u_{m}^{j}}\left(t_{0}\right)$ sampled at *t*_0_ as its columns, is evaluated as a measure of the generalization capability of the liquid for unseen inputs. Unlike *SP*_*lin*_, lower rank of *M*_*a*_ suggests better generalization.

Both AP and SP are important in measuring the computational quality of a liquid. For example, very high quantitative measure for SP and very low measure for AP is ideal. If one liquid has very high SP and a mediocre AP, it is hard to decide whether the particular liquid is better than another liquid with mediocre SP and a very small AP. Therefore, in order to compare the quality of different liquid configurations, a combined measure that involves both SP and AP is required. To address this, we use some insights from Fisher's Linear Discriminant Analysis (LDA) (Fisher, 1936; Fukunaga and Mantock, 1983; Hourdakis and Trahanias, 2013). LDA is utilized to find a linear combination (*f*(.)) of *d* features that characterizes or separates two or more classes (ω_*i*_) of objects. The linear combination as shown in the equation below can be used as a classifier, or as a dimensionality reduction method before classification.

(5) $$\begin{array}{r} y_{i}=f\left(x_{i}\right)=Wx_{i} \end{array}$$

where *y*_*i*_ is the output vector ($Y=\left[y_{1},\ldots ,y_{n}\right]\in {\mathbb{R}}^{L\times n}$), that corresponds to the set of input features, *x*_*i*_ ($X=\left[x_{1},\ldots ,x_{n}\right]\in {\mathbb{R}}^{d\times n}$, each feature *x*_*i*_ is a column vector), *W* ∈ ℝ^*L* × *d*^ is the weight matrix that describes the linear relationship, *L* is the number of classes, and *n* is the number of data samples. The projection from LDA maximizes the separation of instances from different classes, and minimizes the dispersion of data from the same class, simultaneously, to achieve maximum class discrimination. The approximation capability is quantified by the matrix *S*_*w*_ called the “within class scatter matrix” that is specified by

(6) $$\begin{array}{r} S_{w}=\overset{L}{\underset{i=1}{\sum }}P\left(\omega_{i}\right){\hat{\Sigma }}_{i} \end{array}$$

where *P*(ω_*i*_) is the probability of class ω_*i*_, ${\hat{\Sigma }}_{i}$ is the sample covariance matrix (Park and Park, 2018) for class ω_*i*_. The separation capability is given by the “between class scatter matrix” (*S*_*b*_) that is described by

(7) $$\begin{array}{r} S_{b}=\overset{L}{\underset{i=1}{\sum }}P\left(\omega_{i}\right)\left(\mu_{i}-\mu_{g}\right){\left(\mu_{i}-\mu_{g}\right)}^{T} \end{array}$$

where μ_*i*_ is the sample mean vector (centroid) of class ω_*i*_, and μ_*g*_ is the global sample mean vector. In classical LDA, the optimum weight matrix can be found by maximizing the objective function called Fisher's Discriminant Ratio (FDR) (Fukunaga and Mantock, 1983) that is computed as

(8) $$\begin{array}{r} FDR=tr\left(S_{w}^{-1}S_{b}\right) \end{array}$$

where *tr*(.) is the trace operation. For this work, the capability of the liquid to produce a good representation of the input data is quantified by a variant of the above ratio. The FDR is applied on the states of the liquid. However, when the data dimensionality (number of liquid neurons) is large in comparison to the sample size (*n*), the aforementioned scatter matrices tend to become singular (Ji and Ye, 2008) and the classical LDA cannot be applied. Hence, we use a modified discriminant ratio (*DR*) given by the following function:

(9) $$\begin{array}{r} DR=tr\left(S_{b}\right)tr{\left(S_{w}\right)}^{-1} \end{array}$$

**Figure 3:**
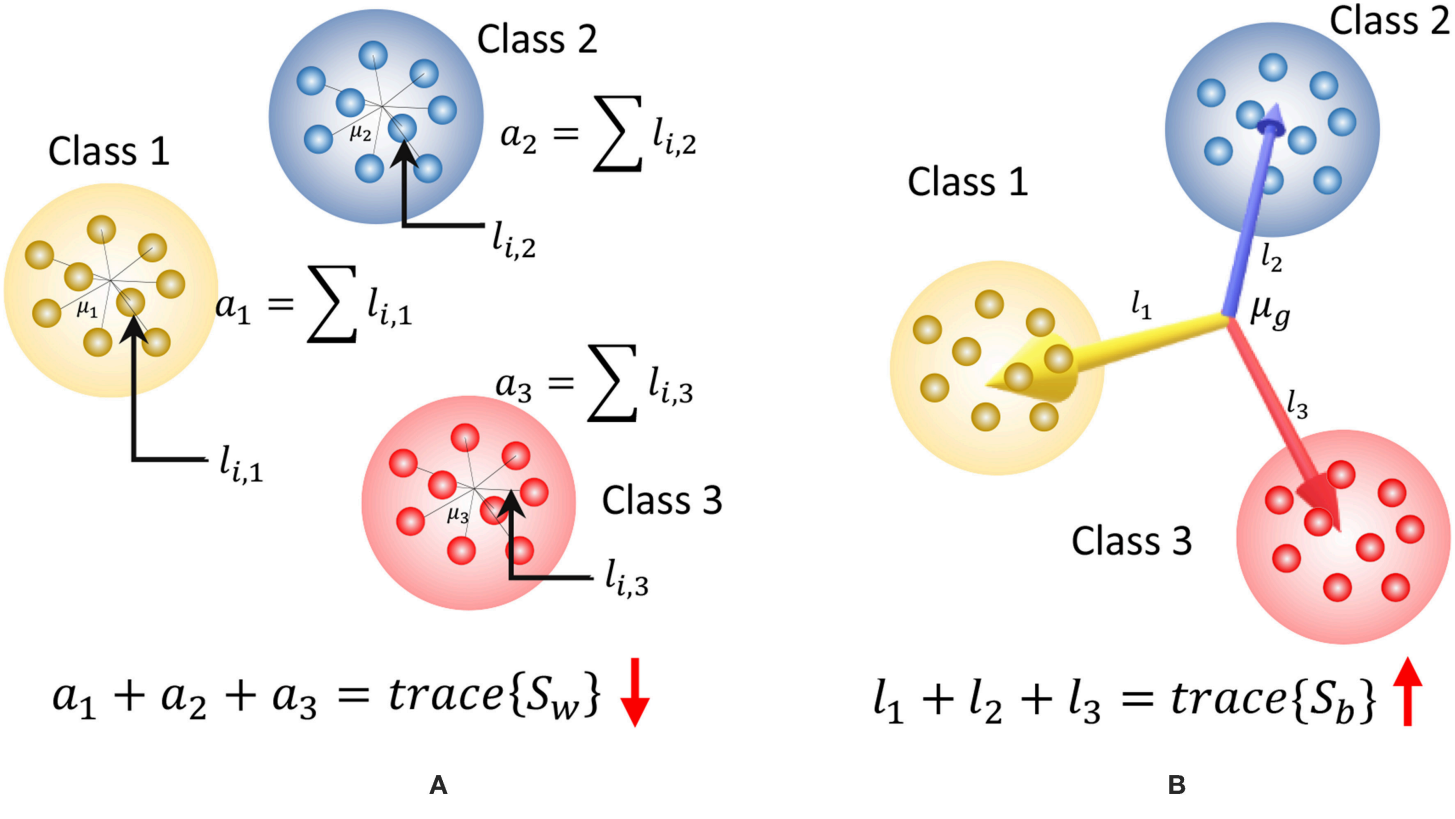
The graphical representation of the components of the discriminant ratio (*DR*) for a set of two dimensional data points that belongs to three classes. **(A)** *tr*(*S*_*w*_) gives a measure of the addition of all the squared distances from the class means to each data point. This must be lower to have better approximation property. Here *l*_*i, j*_ denotes the squared distance between the *i*th data point in class *j* to the class centroid, μ_*j*_ **(B)** *tr*{*S*_*b*_} gives a measure of the addition of the squared distances between the global mean and each class mean. High value for *tr*{*S*_*b*_} signals higher separation property. Here *l*_*i*_ denotes the squared distance from the global mean μ_*g*_ to the centroid of class *i*.

Note that the trace of *S*_*w*_ measures the closeness of each liquid state to its corresponding class mean as illustrated in Figure 3A, and the trace of *S*_*b*_ measures the distance between each class centroid and the global centroid in multidimensional space as depicted in Figure 3B. High *tr*(*S*_*b*_) suggests high SP (hence better) and smaller *tr*(*S*_*w*_) suggests better AP.

### 2.4. Experimental Setup

The performance of the ensemble of liquids approach is compared against a single liquid baseline detailed in section 2.1, with the aid of two spatial image recognition applications and two spatio-temporal speech recognition applications. The liquid was modeled in BRIAN (Goodman and Brette, 2008), a Python-based spiking neural network simulator, and the spiking activities of the neurons were recorded to calculate the liquid state vectors. The state vectors corresponding to the training input instances of each data set were then used to train a single fully-connected classification layer using the stochastic gradient descent algorithm (Robbins and Monro, 1985; Mei et al., 2018). The accuracy of the trained network was calculated on the testing data sets.

#### 2.4.1. Data Sets Used for Illustration

The two spatio-temporal (speech) data sets used in this work are:

1. Digit sub-vocabulary of the TI46 speech corpus (Liberman et al., 1993) (TI-10)
2. TI 26-word “alphabet set”; a sub-vocabulary in the TI46 speech corpus (Liberman et al., 1993) (TI-alpha)

TI-10 consists of utterances of the words “zero” through “nine” (10 classes) by 16 speakers. There are 1, 594 instances in the training data set and 2, 542 instances in the testing data set. TI-alpha, on the other hand, has utterances of the words “A” through “Z” (26 classes). There are 4, 142 and 6, 628 instances in the training and testing data sets, respectively. For the spatial data sets (images), we used the handwritten digits from the MNIST (Deng, 2012) data set containing 60, 000 images of digits 0 through 9 in the training set and 10, 000 images in the testing set. In addition, we also created an extended MNIST data set that contains all the images from the original MNIST data set, and the same set of images transformed by rotation, shifting, and noise injection. It has 240, 000 images in the training data set and 40, 000 images in the testing data set.

#### 2.4.2. Input Spike Generation

The first step is converting the images or the analog speech signals to spike trains to be applied as inputs to the liquid. For spatial data (images), there are *p* number of input spike trains fed in to the liquid, with *p* being the number of pixels in an image. The mean firing rate of each spike train is modulated depending upon the corresponding pixel intensity. Each input image pixel (*i*th pixel) is mapped to a Poisson distributed spike train with the mean firing rate (*r*_*i*_ for the *i*th image pixel) proportional to the corresponding pixel intensity (*I*_*i*_) that is specified by

(10) $$r_{i}=\frac{S_{count,i}}{T}\propto \left(\frac{I_{i}}{255}\right)$$

where *S*_*count, i*_ is the number of spikes generated by the *i*th input neuron within a time period of *T*. For example, mean firing rate in this work for a white pixel (pixel intensity *I*_*i*_ = 255) is selected as 63.75 Hz. For a black pixel (pixel intensity *I*_*i*_ = 0), the mean firing rate is 0Hz. Each image is presented to the liquid for a duration of 300 ms (= *T*).

For the speech data, the audio samples available in wave format were preprocessed based on Lyon's Passive Ear model (Lyon, 1982) of the human cochlea, using Slaney's MATLAB auditory toolbox (Slaney, 1998). The model was used to convert each audio sample to temporal variation in the intensity of 39 frequency channels. These intensity values at each time step *j* (*I*_*i, j*_) were then normalized and used as the instantaneous firing probability of an input neuron *i* (*i* = {1, 2, …, 39}). The time step in this work is 0.5ms.

## 3. Results

### 3.1. The Kernel Quality Improvement Due to the Ensemble Approach

In this section, we will explore the effects of dividing a large liquid, by means of standard measures for SP and AP explained in section 2.3. We involve the same general tasks suggested in Maass et al. (2005); Roy and Basu (2016), to compare the SP and AP. In order to measure the pointwise separation property, we generated 100 input spike trains *u*(*t*) and *v*(*t*) with different distances $d_{u,v}^{in}$ between them. The distance between two input spike trains is evaluated according to the methodology explained in Maass et al. (2005). The two spike trains were first filtered with a Gaussian kernel $e^{-{\left(t/\tau_{in}\right)}^{2}}$, and then the Euclidean distance between them were measured. τ_*in*_ was selected as 5*ms* (Maass et al., 2002).

(11) $$\begin{array}{r} d_{u,v}^{in}=\frac{\left||u\left(t\right)*e^{-{\left(t/\tau_{in}\right)}^{2}}-v\left(t\right)*e^{-{\left(t/\tau_{in}\right)}^{2}}|\right|}{T} \end{array}$$

**Figure 4:**
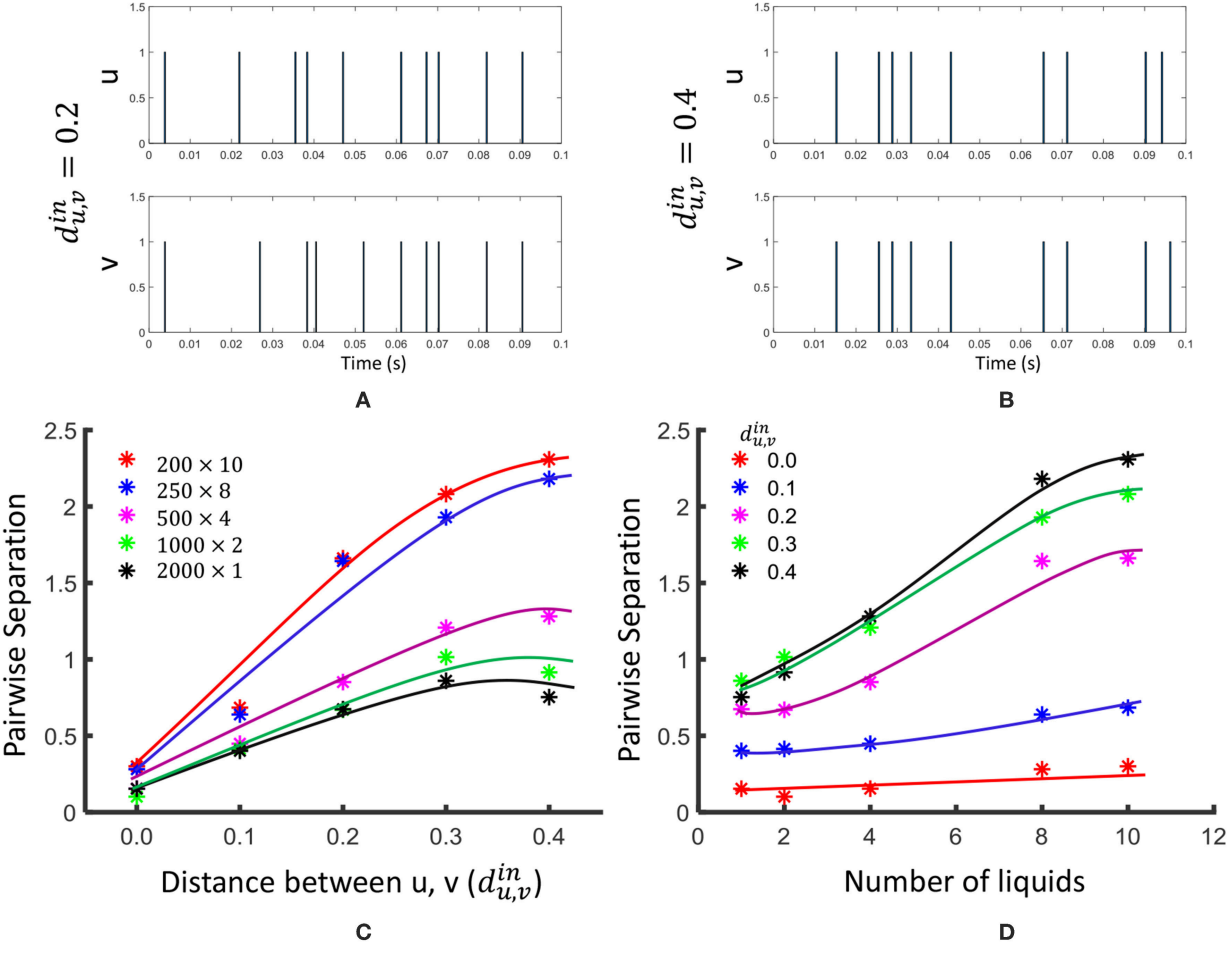
The effect of dividing a large liquid on *SP_pw_*, at different distances $d_{u,v}^{in}$ between inputs. Two input spike trains *u* and *v* are illustrated at **(A)** $d_{u,v}^{in}$ and **(B)** $d_{u,v}^{in}$. **(C)** The variation of pairwise separation with the distances between inputs, at different number of liquids **(D)** The variation of pairwise separation with the number of liquids, at different input distances $d_{u,v}^{in}$.

The same 100 *u*(*t*) and *v*(*t*) signals were fed in to LSMs with different number of liquids (*N*_*ens*_ = 1, 2, 4, 8, 10), and the pairwise separation property was calculated according to Equation 3. The average *SP*_*pw*_ was evaluated over 10 different weight initialization trials and the results are shown in Figure 4. As the figure illustrates, the *SP*_*pw*_ improves with the distance $d_{u,v}^{in}$ between two inputs, and also with the number of liquids in the LSM.

**Figure 5:**
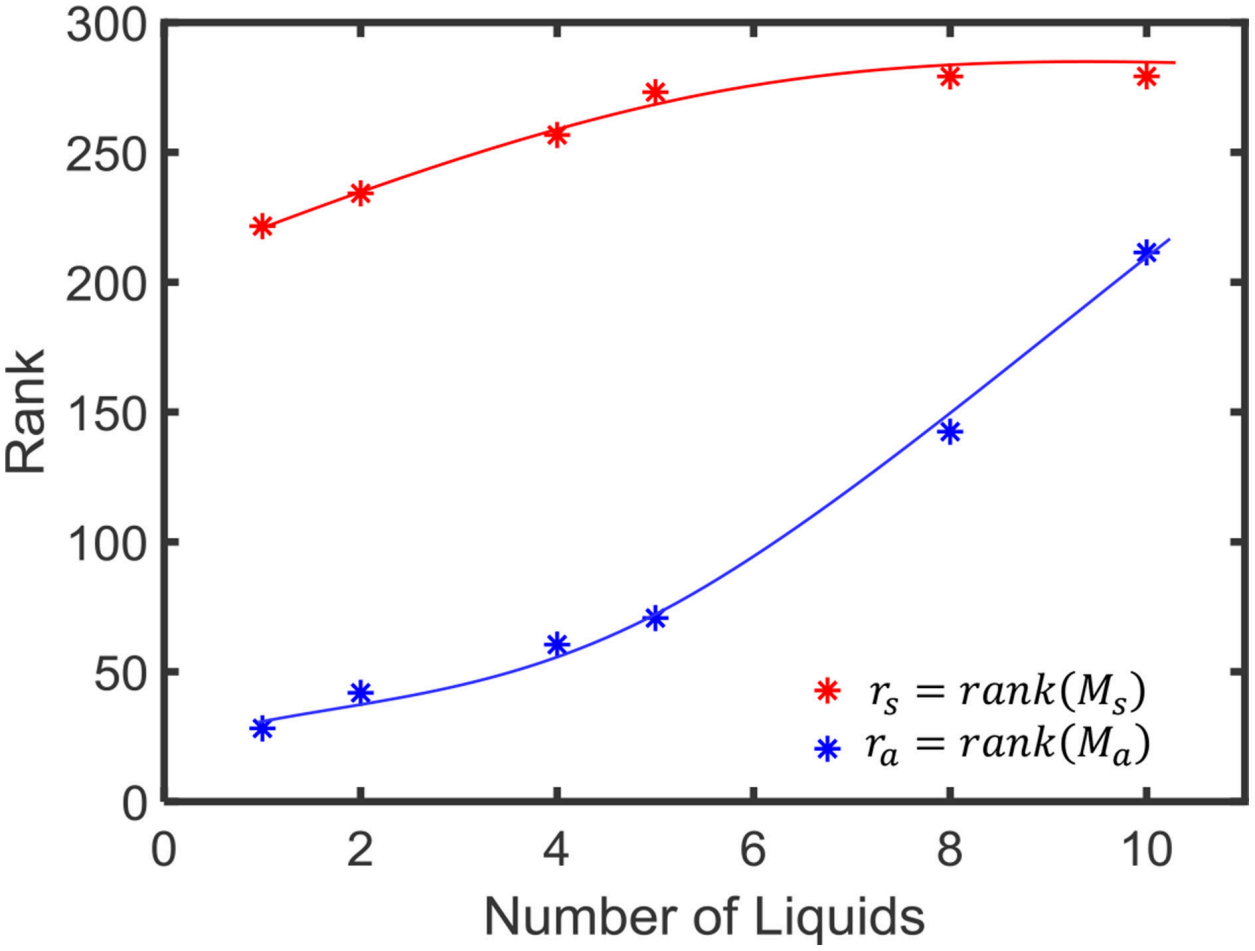
The average rank of state matrix *M*_*s*_ that indicates the inter-class separability (in red) and the average rank of the matrix *M*_*a*_ which is an indication of the intra-class generalization capability (in blue).

For the linear separation property, we applied 400 randomly generated input signals *u*_*i*_(*t*) to LSMs with different number of liquids (*N*_*ens*_ = 1, 2, 4, 8, 10). The resultant states (*x*_*u*_*i*__(*t*_0_)) were used to create the matrix *M*_*s*_ explained in Equation 4. The average *SP*_*lin*_ (= *rank*(*M*_*s*_) = *r*_*s*_) was evaluated among five different sets of inputs and five different weight initializations (i.e., 25 trials altogether) and the results are finalized in Figure 5. As the figure illustrates, the *SP*_*lin*_ increases with the number of liquids. However, the rate of increment of *SP*_*lin*_ reduces with the increasing number of liquids.

For the generalization property, we conducted same above experiment with a different state matrix *M*_*a*_. To create this matrix, we involved 400 jittered versions of the input signal *u*_*i*_(*t*), ($u_{i}^{j}\left(t\right)$) as explained in 2.3. In order to create a jittered version of *u*_*i*_(*t*), we shifted the spike times by a small delay Δ*t* taken from a Gaussian distribution as explained in Maass et al. (2002). The average rank of the matrix *M*_*a*_ is shown in Figure 4. A lower rank of *M*_*a*_ (*r*_*a*_) suggests better approximation of intra-class input examples. According to the figure, *r*_*a*_ increases with the number of liquids. This signals the liquid losing its ability to generalize intra-class inputs.

We observed that the *SP*_*pw*_ improves by 3.06 in the 10-liquid ensemble approach, when comparing with the single liquid baseline. The *SP*_*lin*_ improvement is 1.26×. For a similar set of experiments (for *N*_*tot*_ = 135), the authors in Roy and Basu (2016) explored the kernel quality of an LSM of which the reservoir connections were trained using a structural plasticity rule (Roy and Basu, 2017). The reported improvement in *SP*_*pw*_ is 1.36×, whereas the improvement in *SP*_*lin*_ is 2.05× when compared with a randomly generated traditional LSM. It is noteworthy that when training using structural plasticity, the inter-class separation capability can be improved, with respect to a traditional liquid with random connections. Without involving such complex learning techniques, one can obtain improved separation by simply dividing a liquid as shown in our work. However, note that such reservoir connection learning methods can simultaneously preserve the ability of the LSM to approximate intra-class examples, which is not attainable by the ensemble approach, at higher number of liquids. As explained in section 2.3, the ability of a liquid to produce a better representation of an input is a measure of both SP and AP. In the next section, we will explore this combined measure of SP and AP defined as *DR* in section 2.3, on real world spatio-temporal data classification tasks.

### 3.2. Impact of the Ensemble Approach on Accuracy of Different Applications

**Figure 6:**
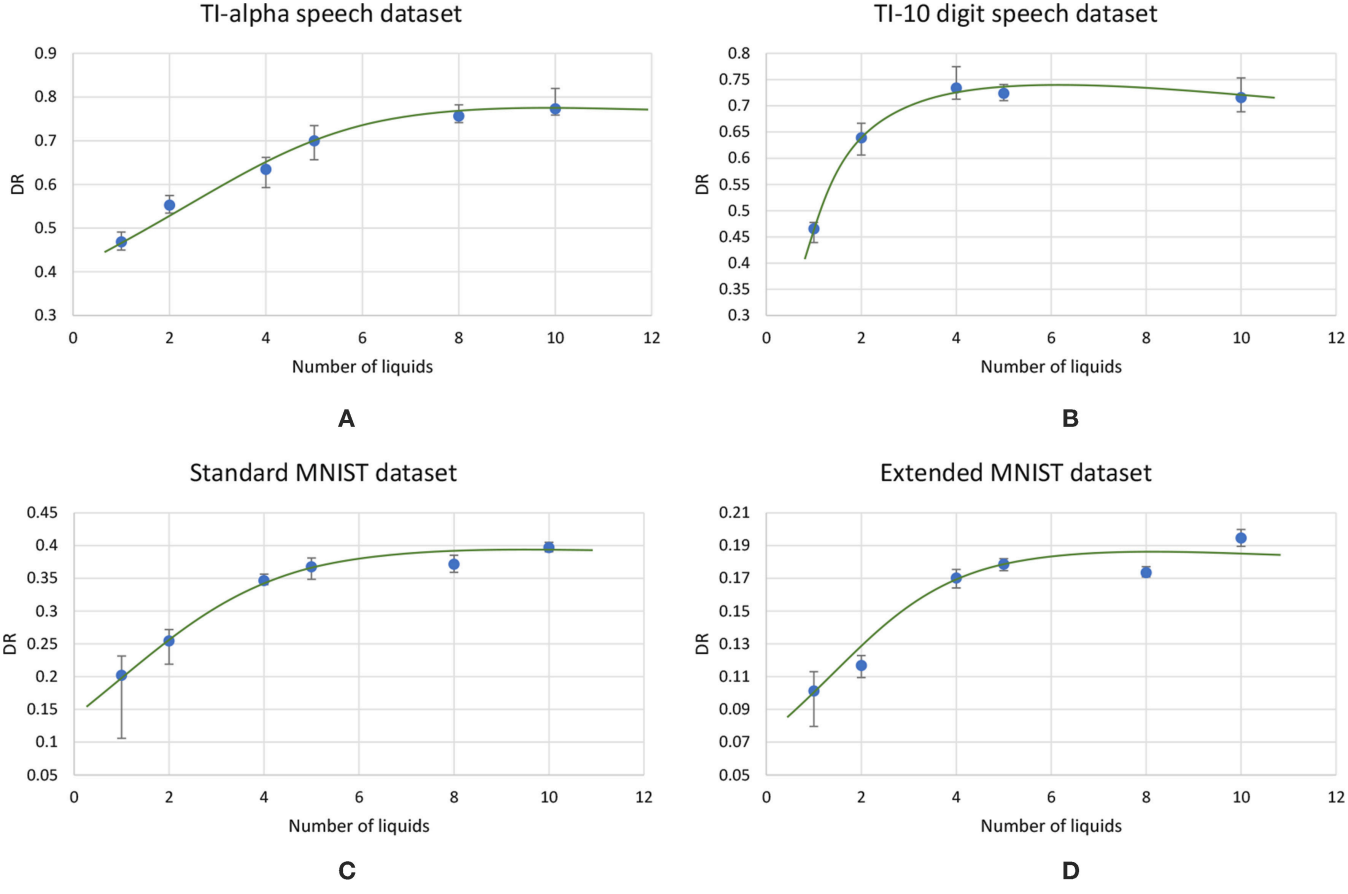
The average (over five trials) discriminant ratio (*DR*) trends with different number of liquids in an ensemble for two speech recognition tasks; **(A)** TI-alpha dataset, **(B)** TI-10 dataset, and two image recognition tasks; **(C)** standard MNIST dataset, **(D)** extended MNIST dataset. The total number of neurons in each ensemble of liquids were kept the same. Note that all the *DR* trends increase with the number of liquids, and saturates after a certain point, that depends on the application.

**Figure 7:**
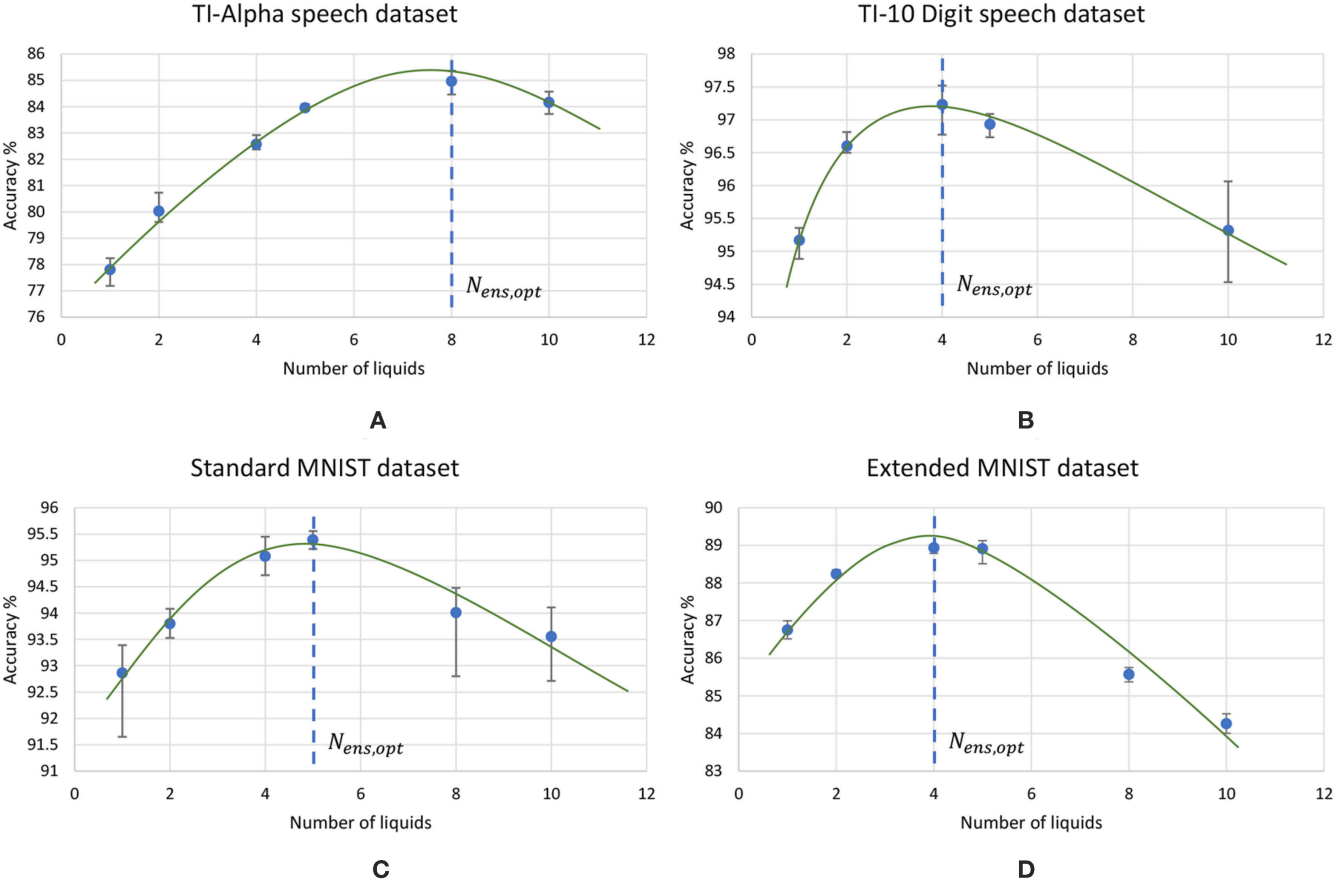
The average (over five trials) accuracy (percentage) trends with different number of liquids in an ensemble, for two speech recognition tasks; **(A)** TI-alpha test dataset, **(B)** TI-10 test dataset, and two image recognition tasks; **(C)** standard MNIST test dataset, **(D)** extended MNIST test dataset. The total number of neurons in each ensemble of liquids were kept the same. Note that all the accuracy trends peak at a certain point, that depends on the application.

Using the experimental setup explained in the previous section 2.4, we initially simulated our baseline single liquid LSM (section 2.1) for the four data sets. We used 500, 2, 000, 1, 000, and 1, 000 neurons in total within the liquid for the TI-10, TI-alpha, standard MNIST, and extended MNIST pattern recognition tasks, respectively. We refined the percentage connectivity for each task as shown in Table 1. The classifier was trained using the liquid states corresponding to the training examples, and the classification accuracy of the trained network was obtained for unseen instances from the test data set. For each application, we then created an ensemble of liquids with $\frac{N_{tot}}{N_{ens}}$ number of neurons in each small liquid. For all the four applications, we evaluated the SP and AP for different number of liquids in the ensemble (*N*_*ens*_ = 1, 2, 4, 5, 8, 10) and quantified how good is the input representation of the ensemble of liquids using *DR* (explained in section 2.3). Figure 6 shows that the *DR* increases up to a certain number of liquids in the ensemble and then saturates for the four different applications we have considered. This signals that the ensemble of liquids, in principle, gives a better representation of the input with increasing number of liquids until a certain point. In order to verify whether this improvement in the *DR* actually implies an improvement in classification accuracy, we evaluated the LSM accuracy for different number of liquids (*N*_*ens*_ = 1, 2, 4, 5, 8, 10) for the four different classification applications. Figure 7 shows that the accuracy indeed improves with the number of liquids until a certain point. Let us denote this point as the “peak accuracy point” and the corresponding number of liquids for that point as the “optimum number of liquids” (*N*_*ens, opt*_). We noticed that the *N*_*ens, opt*_ is a function of the application, and that increasing *N*_*ens*_ beyond *N*_*ens, opt*_ actually results in accuracy loss. When comparing Figures 4, 7, it is evident that the point at which the *DR* saturates is the same as *N*_*ens, opt*_. This explains that dividing a large liquid into multiple smaller liquids enhances the class discrimination capability of the liquid, leading to improved classification accuracy. However, note that after the *N*_*ens, opt*_ point, the *DR* saturates whereas the accuracy degrades. The *DR* does not offer a direct mapping of the accuracy of an LSM. However, it could still be utilized as a measure of identifying the point at which the accuracy starts to drop (*N*_*ens, opt*_). This is the same point at which the *DR* stops improving.

**Figure 8:**
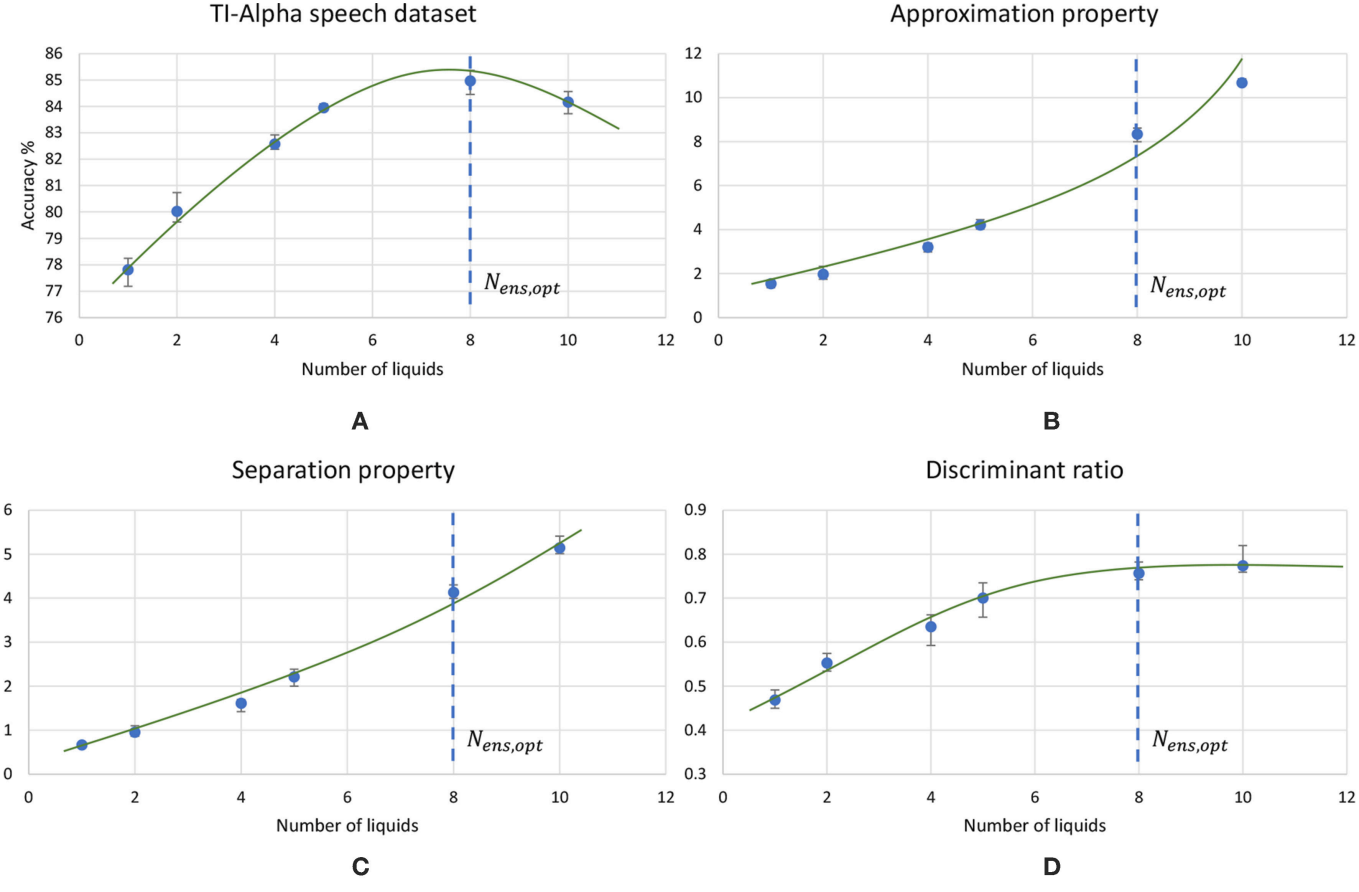
The trends of different measures associated with LSMs, with the increasing number of liquids. **(A)** Accuracy, **(B)** Approximation property (AP), **(C)** Separation property (SP) and **(D)** Discriminant ratio (*DR*). The LSM is trained for TI-alpha speech recognition application. Both AP and SP continuously increases with the number of liquids. Note that the increment in AP is more significant than that of SP for larger number of liquids.

**Figure 9:**
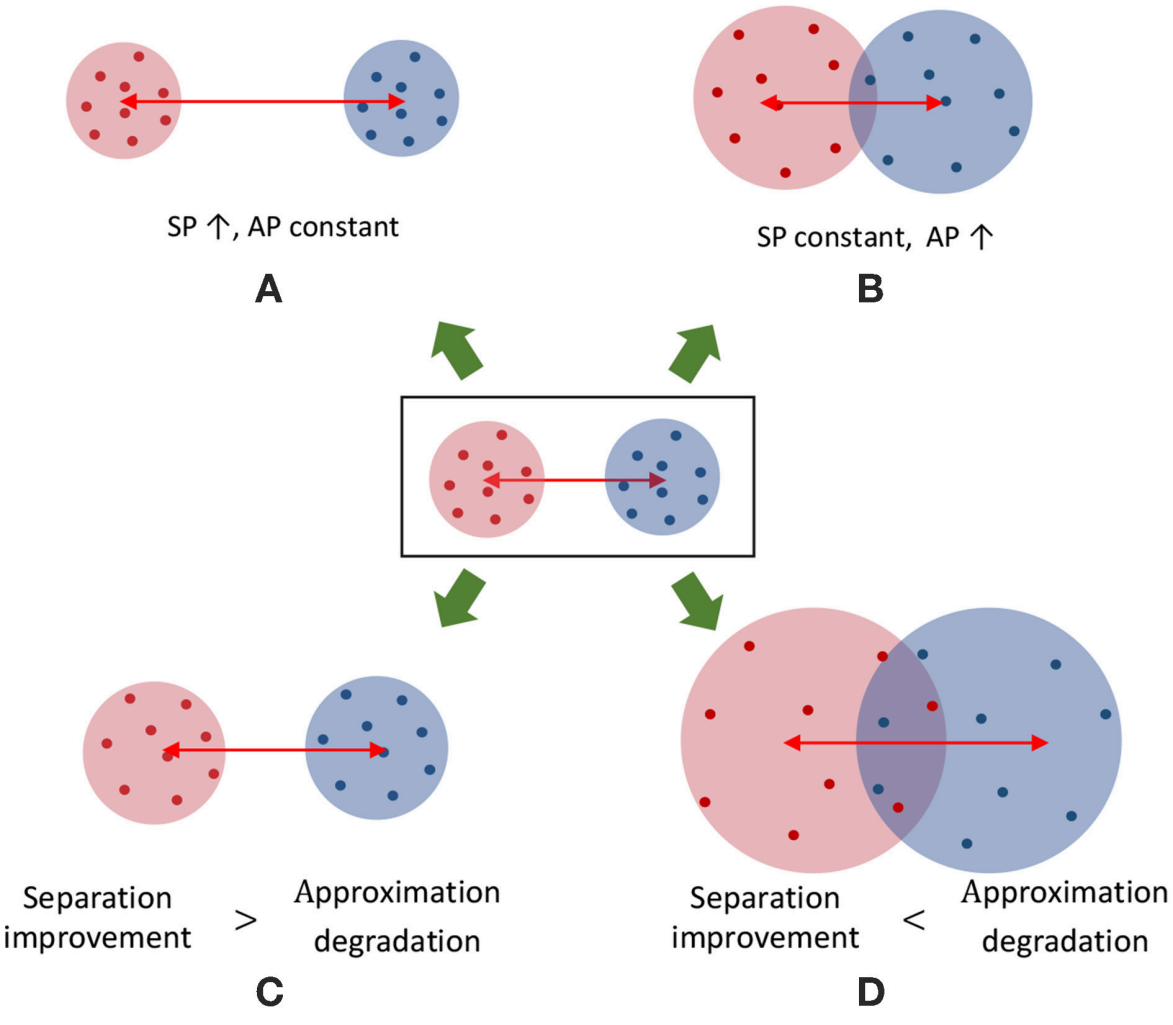
A cartoon that shows the distribution of two dimensional data points that belong to two classes under different conditions. **(A)** A case with increased SP while maintaining the same AP. **(B)** A case where the AP is increased while maintaining the same SP. Note that the class boundaries can get overlapped leading to classification errors. Hence, increased AP is not desirable. **(C,D)** shows two scenarios where both SP and AP increased from the baseline distribution of data points (figure in the middle). **(C)** The improvement in SP is larger than the degradation in AP. **(D)** The improvement in SP is not sufficient to compensate for the AP degradation, leading to overlapped class boundaries.

Figure 8 plots the variation of the individual *DR* components; separation (*SP* = *tr*(*S*_*b*_)) and approximation (*AP* = *tr*(*S*_*b*_)) with the number of liquids for the TI-alpha speech recognition task. Figure 8 shows that SP improves continuously with the number of liquids. Improved separation suggests larger dispersion among the centroids of the liquid states corresponding to instances from different classes, which renders the input representations provided by the liquid easier to classify. This is illustrated in the cartoon in Figure 9A for a set of two-dimensional data points from two classes, wherein higher SP while maintaining the same AP results in enhanced class discrimination capability. At the same time, Figure 8 indicates that AP also increases with the number of liquids, implying that larger number of liquids leads to higher dispersion between projected inputs from the same class. Higher AP for a given SP is not desirable since it could potentially lead to overlap among instances belonging to different classes as depicted in Figure 9B, thereby degrading the class discrimination capability. Since both SP and AP increases, the ratio *DR* gives a better measure about the overall effect of the proposed ensemble approach on the classification accuracy of the LSM rather than the individual components *per se*. As shown in Figure 8, the *DR* increases until a certain number of liquids, signaling the dominance of the improvement in SP over the degradation in AP as graphically illustrated in Figure 9C. In contrast, as the number of liquids is increased beyond *N*_*ens, opt*_, *DR* saturates since the increment in SP is no longer sufficient to compensate for the degradation in AP as shown in Figure 8. When the dispersion between classes (due to increment in SP) is not sufficient to compensate for the dispersion occurring for instances within the same class (due to AP degradation), there can be overlaps among class boundaries as depicted in Figure 9D, leading to accuracy loss as experimentally validated in Figure 7 across different applications.

**Figure 10:**
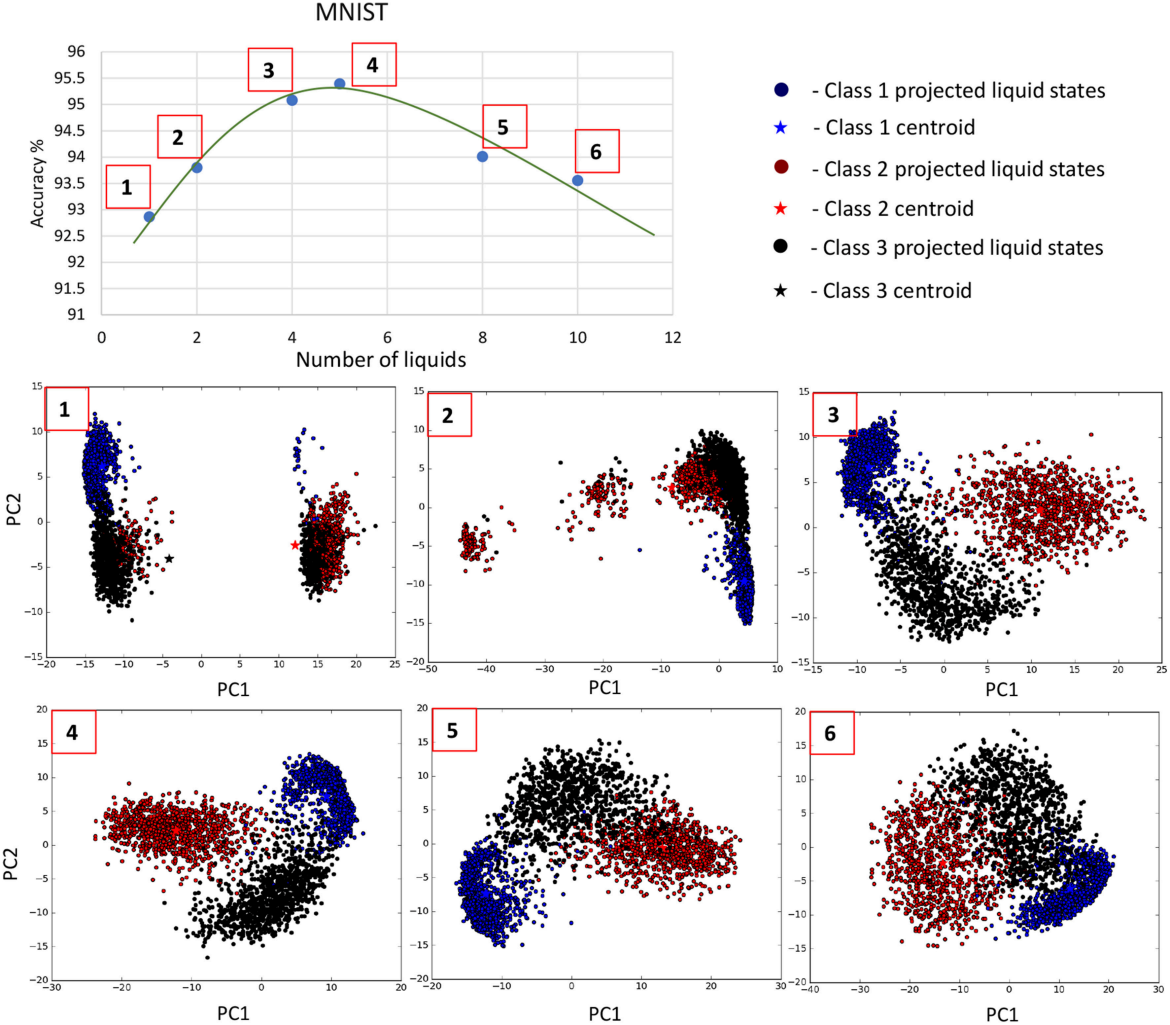
The distribution of the liquid state vectors, as a projection to the first two principal components PC1 and PC2, for different number of liquids. The liquid state vectors (represented as dots) correspond to three classes in the MNIST image data set. Each class has randomly picked 1, 000 liquid state vectors. Distributions related to point 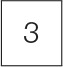 and 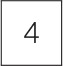 show less overlapping between classes, and the data points are more concentrated at the class mean points in contrast to 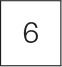, which has significant overlapping that caused the accuracy degradation.

In order to graphically view the variation in SP and AP with the number of liquids for the applications considered in this work, we used Principal Component Analysis (PCA) to plot the high-dimensional liquid states in a low-dimensional space. Generally, the first few principal components preserves the most variance in a given high-dimensional data set. Hence, the same object in multi-dimensional space can be visualized in low-dimensional space with insignificant changes. To create such a low-dimensional projection of the liquid state vectors for different input patterns, we reduced their dimension using PCA and plotted the two most significant Principal Components (PCs) corresponding to the two largest eigenvalues. Figure 10 plots the 800-dimensional liquid state vectors, projected to the two-dimensional space using the first two PCs, for 1, 000 randomly picked input patterns from three classes in the MNIST data set. Figure 10 clearly illustrates why the accuracy improves till the *N*_*ens, opt*_ point and degrades beyond that as explained below. The single liquid case shows concentrated (low AP), but overlapped data (low SP). This is where the AP is the lowest due to the concentrated data points. As the number of liquids increases, the classes become clearly separated. Note that the points belonging to the same class also moves away from their respective centroids due to the increased AP. This ultimately results in the aforementioned overlapping between the classes for number of liquids larger than *N*_*ens, opt*_, which gives rise to more misclassifications.

### 3.3. Benefits of the Ensemble Approach

**Figure 11:**
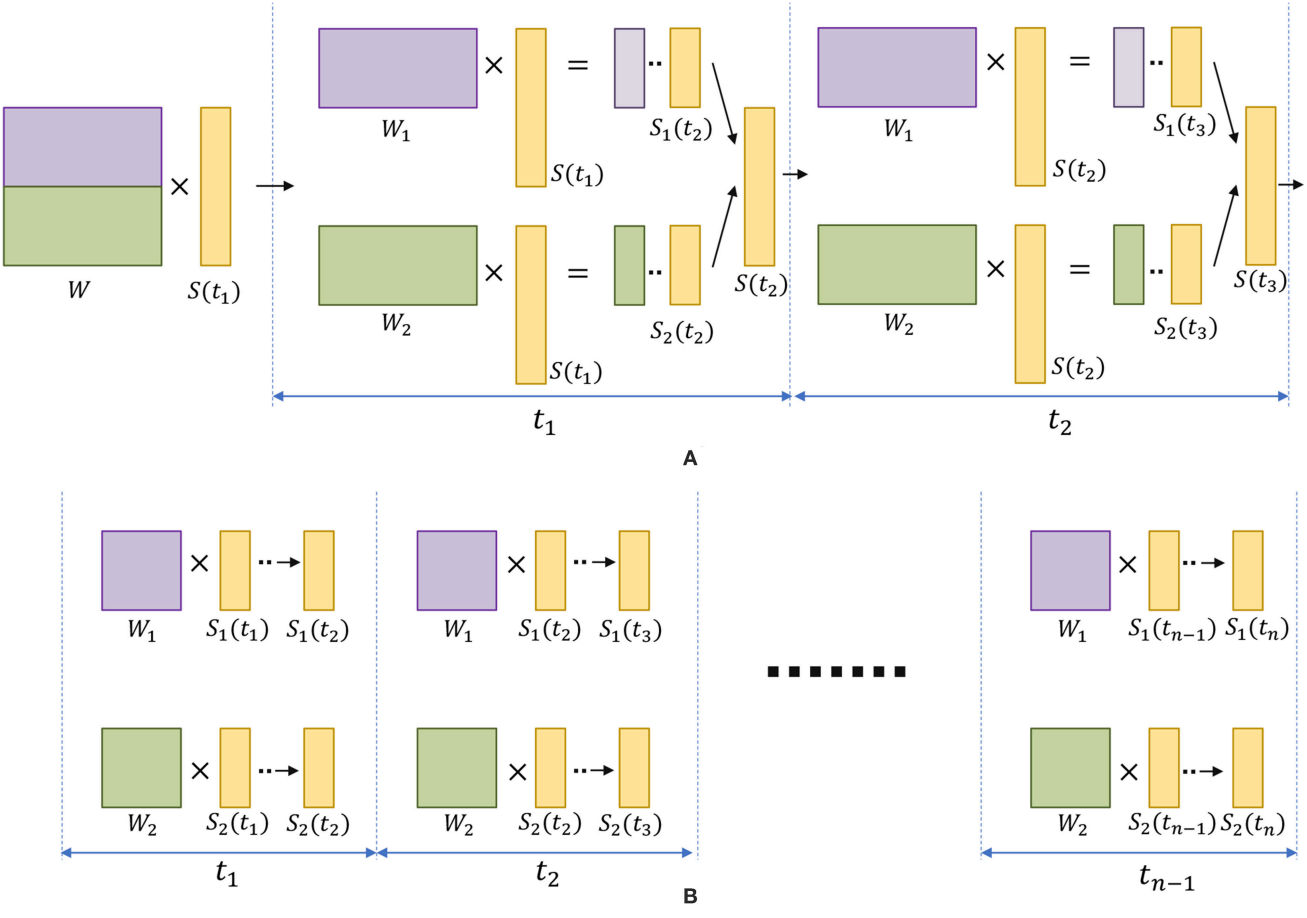
**(A)** The division of matrix-vector multiplication using row-wise striped matrix decomposition, for the single liquid baseline LSM. Note that during each time step, the generated *S*_1_ and *S*_2_ vectors need to be concatenated to form the *S* vector (represents the spiking activity of the liquid), which requires communication between cores. **(B)** The “embarrassingly parallel” nature, and the reduced amount of operations in the ensemble approach allows two small liquids to run in parallel as two independent tasks, until the end of the last simulation time step *t*_*n*_.

The ensemble of liquids approach creates smaller liquids where the dynamics of one network does not affect another. When evaluating the spike propagation within the liquids, these smaller liquids can be run independently and in parallel. Since the evaluation time is a higher order polynomial function of the number of neurons, computing few smaller liquids in parallel instead of computing one large liquid is beneficial in terms of reducing the inference time. Note that the evaluation of a large liquid can also be parallelized. The liquid dynamics vary temporally, and for digital simulations, it can be divided in to multiple time steps. Each evaluated neuron state in the liquid at one time step is temporally correlated to that of the next time step. Therefore, the liquid evaluation process cannot be temporally divided for parallelizing the operation. Furthermore, since all the neurons are connected to each other (with a given sparsity), the dynamics of one neuron is dependent upon that of other neurons connected to it. Therefore, “fully independent” simulations are also not possible at the neuron level. However, the matrix-vector manipulations involved in each time step *can* be parallelized. Simply put, in finding the pre-synaptic currents of the neurons, the matrix-vector multiplication between the spiking activity and the weight matrix must be evaluated as shown below (with respect to excitatory neurons for example).

(12) $$\begin{array}{r} \Delta g_{e}\left(t_{i}\right)=WS\left(t_{i}\right) \end{array}$$

where Δ*g*_*e*_(*t*_*i*_) is the instantaneous jump of conductance at time *t*_*i*_ (refer to Equation 2), *S*(*t*_*i*_) is the spiking activity vector of *N* number of neurons in the liquid at time *t*_*i*_, and *W* ∈ ℝ^*N*×*N*^ is the connection matrix that defines the liquid. Consider dividing the above process in to multiple processing cores. The division of the operation in to two cores using row-wise striped matrix decomposition requires the matrix *W* to be divided in to two parts (Figure 11A). During each simulation time step (*t*_*i*_), each core evaluates membrane potentials [*S*_1_(*t*_*i*+1_) =*s*_1_(*t*_*i*+1_), …, *s*_*N*/2_(*t*_*i*+1_)] and [*S*_2_(*t*_*i*+1_) =*s*_*N*/2+1_(*t*_*i*+1_), …, *s*_*N*_(*t*_*i*+1_)]. For the next time step, these *S*_1_ and *S*_2_ must be concatenated and requires communication between cores. In contrast, a concatenation is not required until the end of the total simulation duration (*T*) in our ensemble approach (Figure 11B). Due to the lack of communication overhead between processors, the ensemble approach is faster than a parallelized version of the single liquid baseline among *N*_*ens*_ number of processors. In fact, due to the aforementioned communication overheads, efficient parallel processing can be hindered even in Graphical processing units (GPUs)(Kasap and van Opstal, 2018). However, in any method of evaluating the liquid dynamics, note that the ensemble approach has less number of connections than a single liquid baseline. Therefore, the ensemble approach has reduced amount of computation leading to lower evaluation time. Different studies have shown designing hardware accelerators for spiking neural network platforms (Wang et al., 2014, 2017; Du et al., 2017). In the context of reducing the design complexity, above methods could potentially benefit from the low connection complexity, and “embarrassingly parallel” nature (Herlihy and Nir, 2011) of our ensemble approach.

The inference time is the addition of the liquid evaluation time and the classifier evaluation time. The liquid evaluation time was calculated by giving 100 input instances to the LSM model solver and estimating the average liquid computation time per input. The classifier evaluation time is significantly lower than the liquid computation time (~ × 10). Note that the classifier training time is similar in the baseline (single liquid LSM) and the ensemble approach, since there are equal number of neurons in the liquid and the number of trained weights are the same.

**Figure 12:**
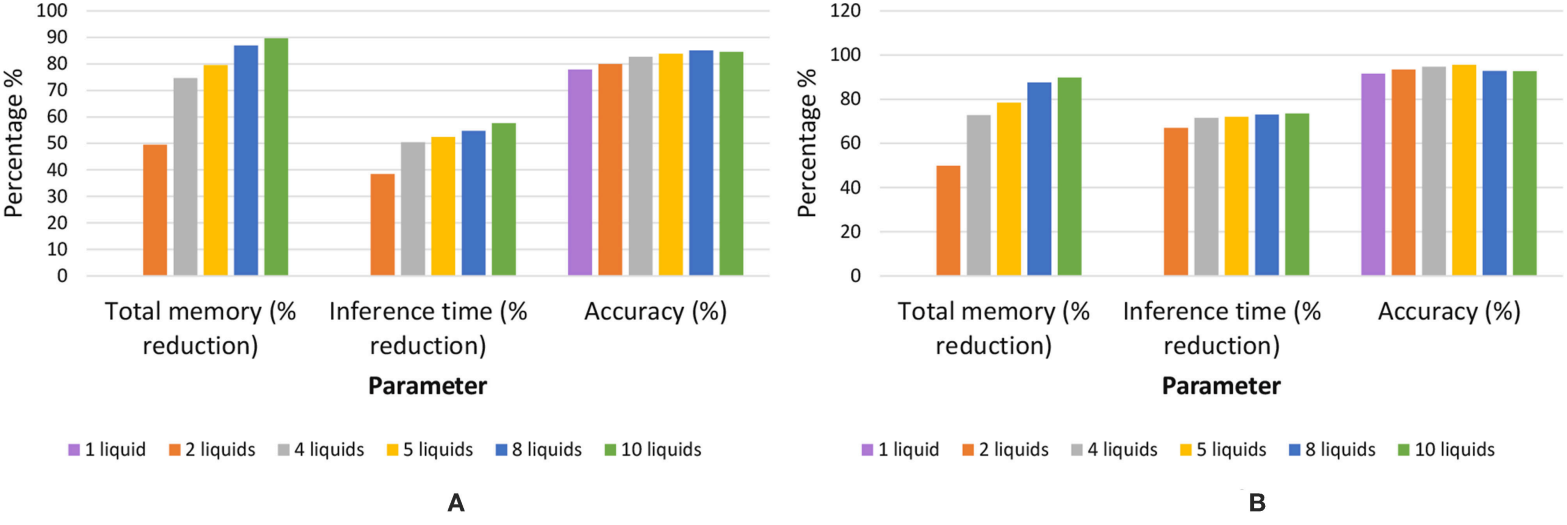
The total memory reduction (%), inference time reduction (%) with respect to the baseline, and accuracy for different number of liquids in the ensemble. Two applications were considered; **(A)** temporal data classification problem (TI-alpha) **(B)** spatial data classification problem (MNIST).

Once an LSM is trained, the connections within the liquid and the classifier weights must be stored. LSMs with large liquids require more space. In the ensemble approach, the number of connections within the liquid are significantly lower than the single liquid baseline. For example, assume dividing a liquid with *N*_*tot*_ number of neurons in to *N*_*ens*_ number of smaller liquids with $\frac{N_{tot}}{N_{ens}}$ amount of neurons in each of them. The number of connections available within the liquid for the single liquid baseline is $\sim N_{tot}^{2}$ whereas the number of connections in the multi-liquid case is $\sim {\left(\frac{N_{tot}}{N_{ens}}\right)}^{2}N_{ens}=\frac{N_{tot}^{2}}{N_{ens}}$. This shows that the number of connections reduces by a factor of *N*_*ens*_ when dividing a large liquid into *N*_*ens*_ smaller liquids, given that the percentage connectivity stays the same. Figures 12A,B illustrate how the memory requirement varies for different number of liquids for the MNIST image recognition and TI-alpha speech recognition applications, respectively. When the optimum accuracy point for the ensemble approach is considered, we witnessed 87% reduction in the amount of memory, 55% reduction in inference time, and a 7.3% improvement in accuracy simultaneously, for the TI-alpha speech recognition application. For the MNIST handwritten digit recognition application, we witnessed 78% reduction in the amount of memory, 72% reduction in the inference time, and 3.9% improvement in classification accuracy.

### 3.4. Conventional Methods of Improving the Accuracy vs. the Ensemble Approach

The simple structure and training of LSMs, come with an accuracy trade-off, when compared with other non-reservoir computing techniques such as LSTM networks (Bellec et al., 2018). Different mechanisms have been studied in the literature such as training the connections in the reservoir (Xue et al., 2016), using expensive learning rules (for example, backpropagation through time Bellec et al., 2018), and selecting complex architectures (Wang and Li, 2016), in order to improve the accuracy of liquid state machines. However, these methods will increase the complexity of the LSM resulting in poor performance with respect to latency, despite the higher accuracy. Furthermore, a liquid can be considered as a universal computational medium. A single liquid with multiple trained readouts can be used for multiple applications(Wang et al., 2015). Above methods such as training the connections within the liquid will make the LSM restricted to one application. In this section, we will explain two basic methods of improving accuracy, while leaving the structural and training simplicity of LSMs intact, and compare the results with the ensemble approach.

#### 3.4.1. Increasing the Number of Neurons in the Liquid

**Figure 13:**
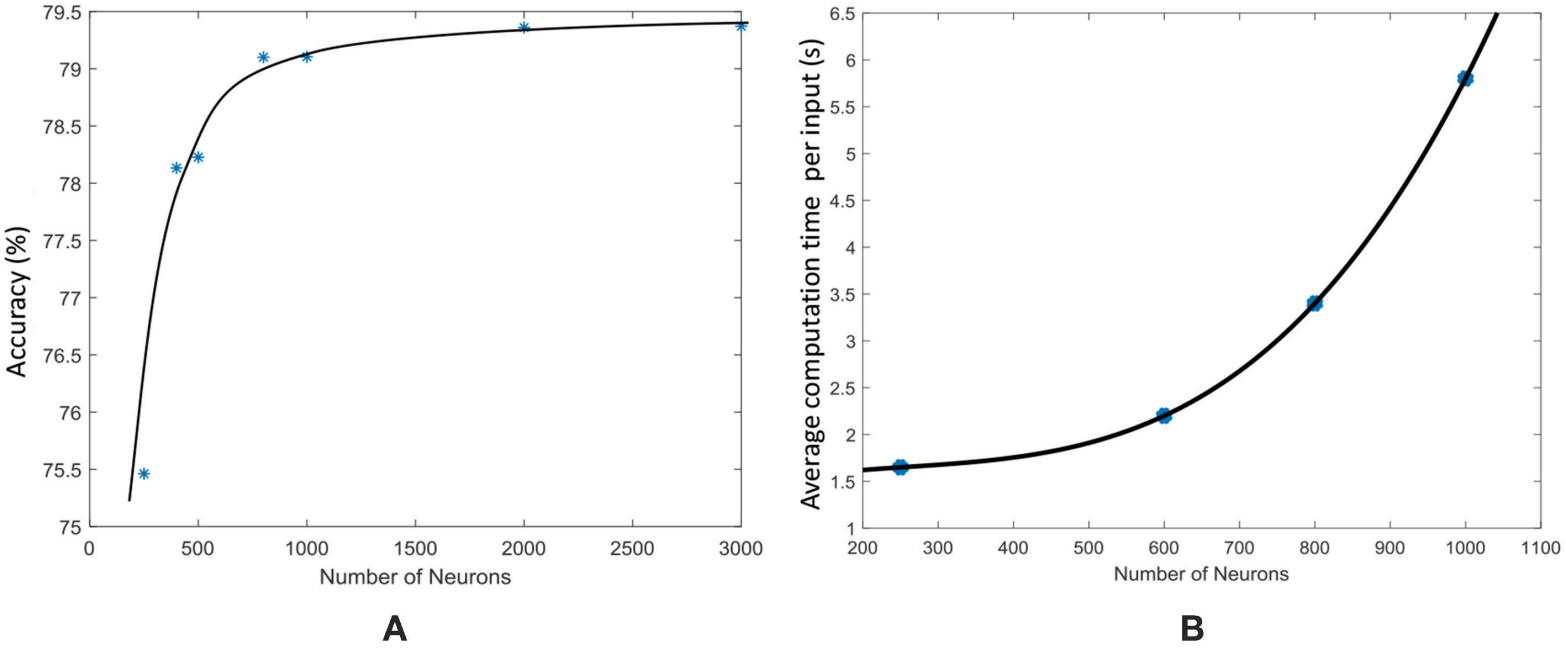
**(A)** The accuracy of an LSM with a single liquid, measured at different number of neurons, for a speech recognition application (TI-alpha). **(B)** The average liquid evaluation time of an LSM measured at different number of neurons.

As explained in Maass et al. (2003), sufficiently large and complex liquids possess large computational power. Increased number of neurons in the liquid will result in increased number of variables for the classifier. Based on “multiple linear regression” methods of predicting a function, increased number of predictor variables (in this case the number of neurons), will result in better prediction (Krzywinski and Altman, 2015; Wijesinghe et al., 2017). Therefore, increasing the number of neurons will improve the prediction accuracy of the LSM. Note however that using enormous number of predictor variables/neurons will make the network suffer from overfitting. Figure 13A shows how the accuracy of an LSM varies with the number of neurons in the reservoir for the TI-alpha speech recognition task. As Figure 13A illustrates, the accuracy initially increases with the number of neurons and then saturates after a certain point. Increased number of neurons implies increased connections within the liquid, given that the percentage connectivity stays the same. The number of connections within the liquid shows a square relationship $\sim \nu N_{tot}^{2}$ with the number of neurons *N*_*tot*_, where ν is the global percentage connectivity. Due to this, evaluation time of the liquid increases exponentially as shown in Figure 13B. Therefore, when the number of neurons are already high, the accuracy improvement we obtain by further increasing the number of neurons is not worth the resultant performance and storage requirement penalty. Furthermore, the accuracy saturates around ~79.2% for the TI-alpha application (for *N*_*tot*_ ≥ 800). Note that we have also adjusted the percentage connectivity at each point in the graph, to get the best accuracy for a given number of neurons. However, the ensemble approach for *N*_*tot*_ = 1000 and *N*_*ens*_ = 4 gives ~83% accuracy, which is larger than the accuracy achievable by increasing the number of neurons in a single liquid.

#### 3.4.2. Percentage Connectivity Within the Liquid

**Figure 14:**
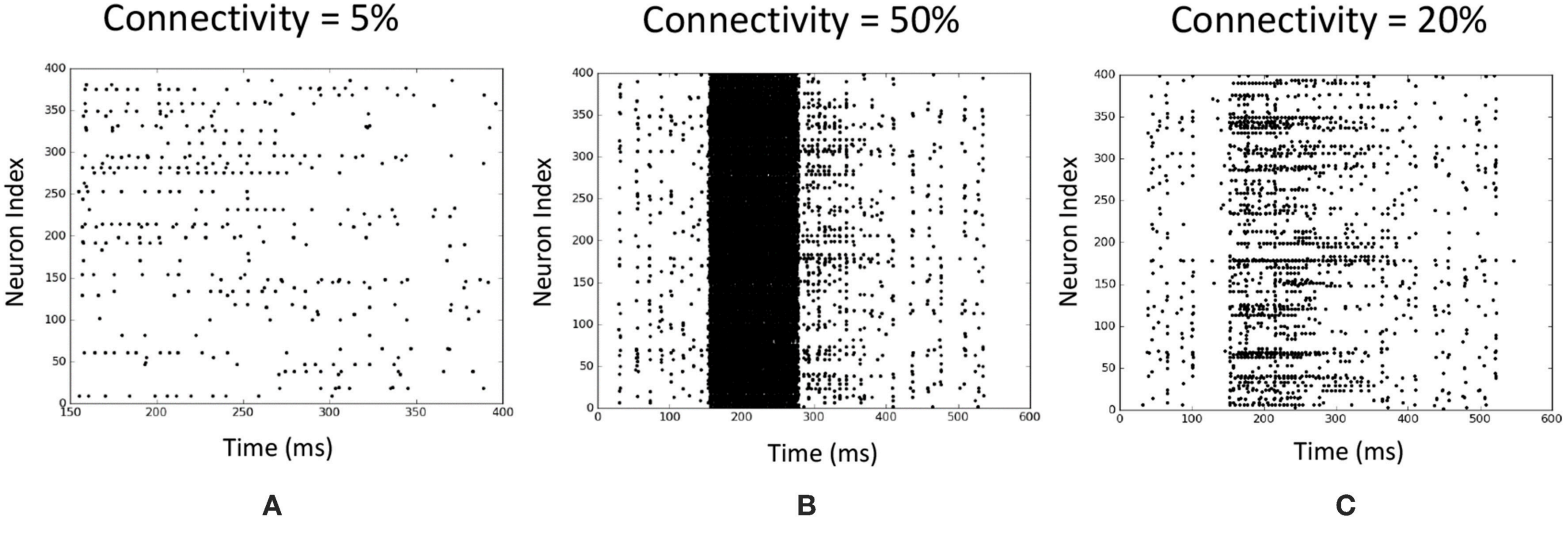
Illustration of two negative behaviors of an LSM at different input to liquid percentage connectivity values. Each raster plot shows the spiking activity of the liquid neurons over time. The application is a speech recognition task (TI-alpha). **(A)** Over-stratification at low percentage connectivity. **(B)** Pathological synchrony at higher percentage connectivity. **(C)** An instance that shows clear differences between spiking activity of the liquid neurons in contrast to **(A,B)**.

The percentage connectivity within the LSM is an important measure of the spiking activity of a liquid. The spiking activity of the liquid could show two negative behaviors which could drastically reduce the accuracy of the network, *viz*. pathological synchrony and over-stratification (Norton and Ventura, 2006). Pathological synchrony occurs when the neurons get caught in infinite positive feedback loops resulting in heavy continuous spiking activity. Over-stratification can be defined as the opposite extreme of the above. Here, the neurons do not propagate an input signal properly, resulting in reduced spiking activity. Both the above behaviors result in similar outcomes for input instances of different classes (hence poor separation between classes), making classification tasks hard. We noticed that lower connectivity (*P*_*In*→*E*_) results in over-stratification (Figure 14A) whereas higher connectivity results in pathological synchrony (Figure 14B).

**Figure 15:**
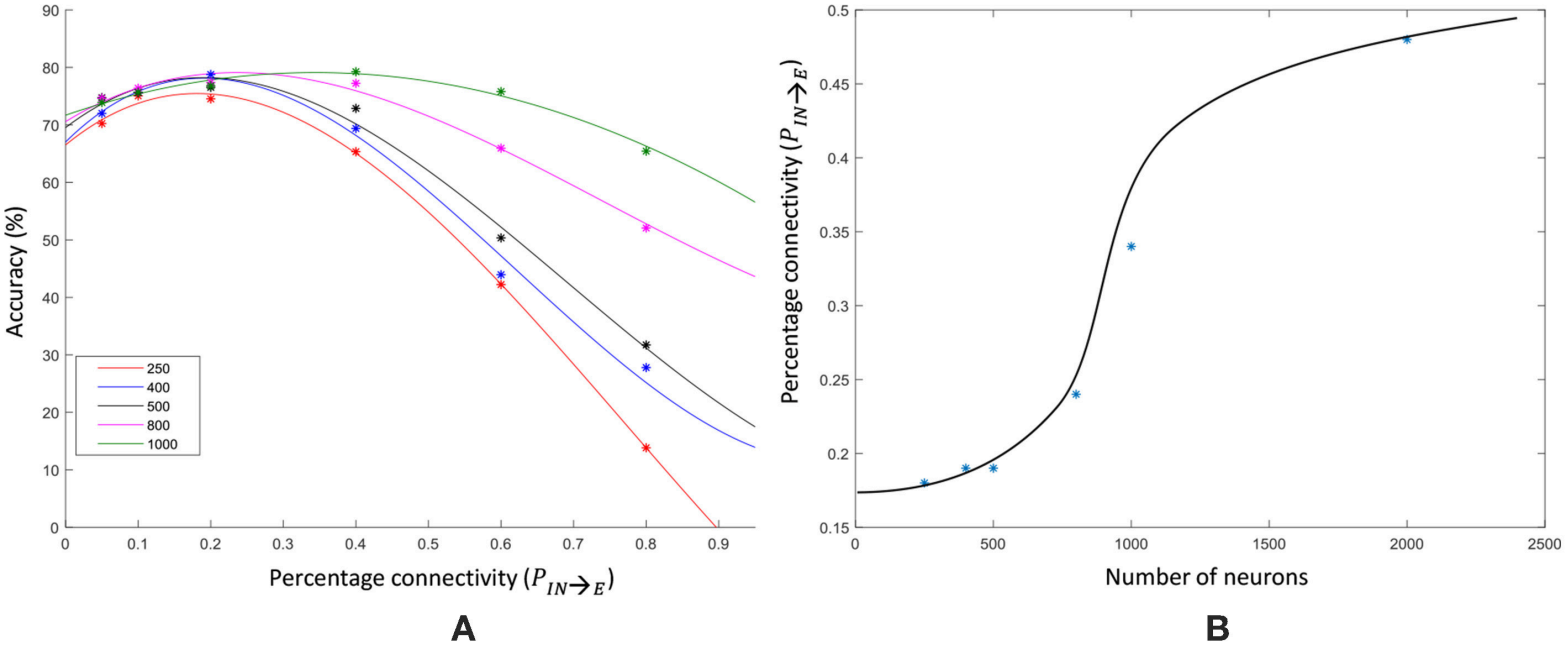
**(A)** The accuracy trend with varying input–liquid percentage connectivity, for different number of liquid neurons. The experiment is done on a single liquid LSM conducting a speech classification task (TI-alpha). **(B)** The percentage connectivity that gives the best accuracy at different number of neurons.

We changed the percentage connectivity between different combinations of pre- and post-neuron types (*E*−*E, I*−*E, E*−*I*) till we obtain good accuracy avoiding pathological synchrony and over-stratification (Figure 14C). After that, we refined the input-liquid connectivity for further accuracy improvement. Figure 15A shows how the accuracy changes with the percentage connectivity of the input to liquid connections. Liquids with different number of neurons have different optimum connectivity values as shown in Figure 15B. The application is recognizing spoken letters in TI-alpha speech corpus. The maximum accuracy achievable by changing the percentage connectivity (*P*_*IN*→*E*_) for *N*_*tot*_ = 1, 000 is ~79% (refer to the green colored trend in Figure 15A). This is smaller than that achievable (~83%) by our ensemble approach with *N*_*tot*_ = 1, 000 and *N*_*ens*_ = 4.

**Figure 16:**
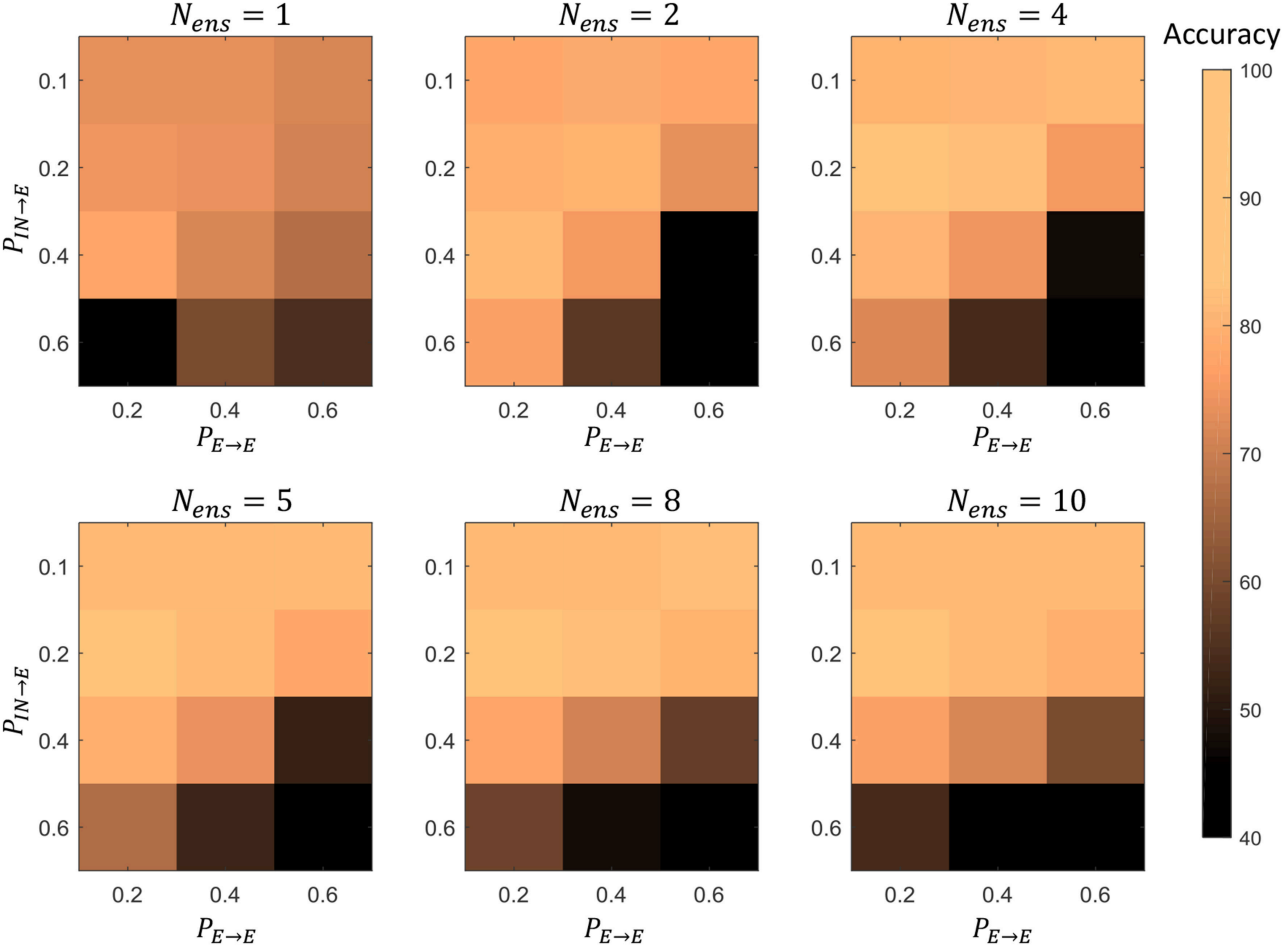
The accuracy of LSMs with *N*_*ens*_ = 1, 2, 4, 5, 8, 10 at different percentage connectivity (*P*_*E*→*E*_ and *P*_*IN*→*E*_) values, for the TI46-alpha classification task.

**Figure 17:**
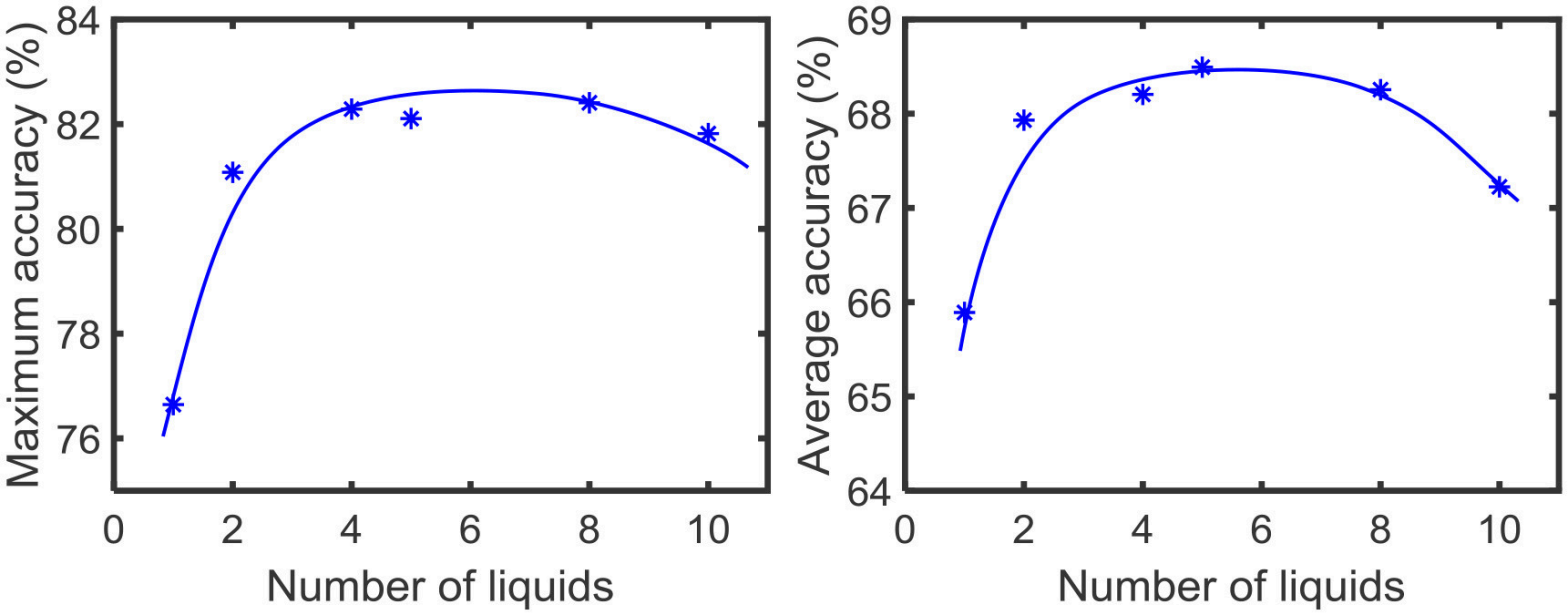
**(A)** The maximum accuracy among all the LSM configurations with different *P*_*IN*→*E*_ and *P*_*E*→*E*_ **(B)** The average accuracy across all the LSM configurations with different *P*_*IN*→*E*_ and *P*_*E*→*E*_.

Furthermore, we simultaneously changed the *P*_*E*→*E*_ and *P*_*IN*→*E*_ percentage connectivity values of LSMs with different number of liquids, and evaluated the accuracy. Four *P*_*IN*→*E*_ values (0.1, 0.2, 0.4, 0.6) and three *P*_*E*→*E*_ values (0.2, 0.4, 0.6) were selected for the experiment. The summarized results are illustrated in the 3D plot in Figure 16. The color code of the figure gives the accuracy of a particular combination of connectivity values. Across all LSM configurations with different number of liquids, we witnessed that higher *P*_*IN*→*E*_ and higher *P*_*E*→*E*_ results in accuracy degradation. Sparser connectivity gives better results. As the figure illustrates, at sparser connectivity values, a single liquid LSM offers lower accuracy than an LSM with *N*_*ens*_ liquids (refer to the upper left corner of the 3D plots). The “maximum capacity” of each LSM configuration (for a given number of liquids) is plotted in Figure 17A. The “maximum capacity” is the best accuracy attainable from a particular liquid configuration, after optimizing the percentage connectivity values (in the selected range). As Figure 17A illustrates, maximum accuracy obtained from the single liquid configuration is smaller than that of other configurations. We also plotted the average accuracy of a given LSM configuration across all percentage connectivity values (Figure 17B). The average accuracy to some extent could be thought of as the outcome one would witness in a given LSM configuration for an arbitrarily selected connectivity value (within the specified sparse connectivity region of the experiment). The average accuracy of the single liquid LSM configuration is lower than that of multiple liquids.

**Figure 18:**
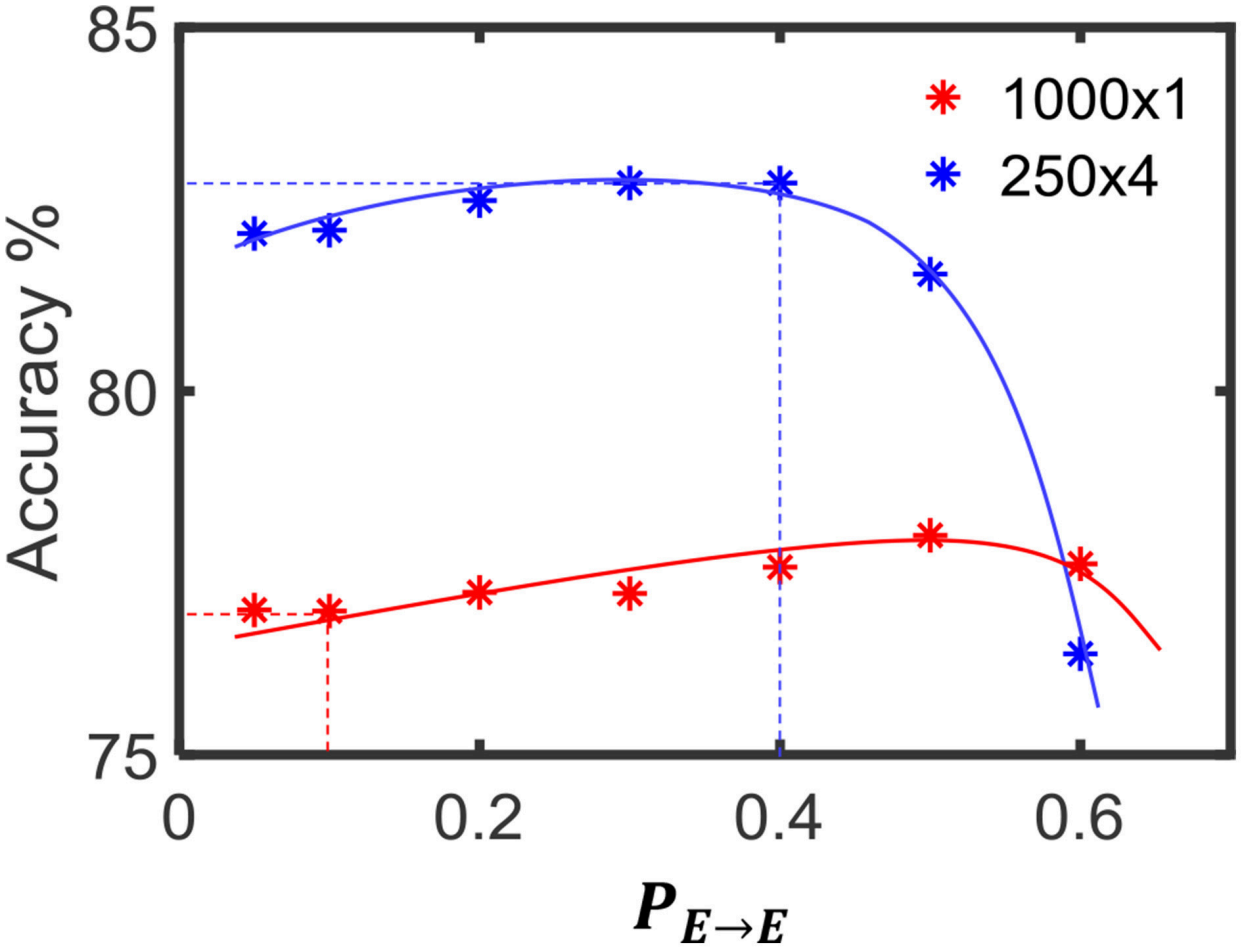
The accuracy variation of the single liquid LSM baseline and ensemble approach (*N*_*ens*_ = 4) at different percentage connectivity values (*P*_*E*→*E*_). The accuracy of the ensemble approach at *P*_*E*→*E*_ = 0.4 is higher than the accuracy of the single liquid LSM at *P*_*E*→*E*_ = 0.1. Note that the number of connections in both the cases considered are the same. The accuracy was evaluated on the TI46-alpha classification task.

In section 3.3, we explored the benefits of the ensemble approach due to reduced number of connections in the liquid. A single liquid LSM configuration has *N*_*ens*_ times more number of connections as the LSM with *N*_*ens*_ number of liquids as explained in section 3.3. In order to view if a single liquid with sparser connectivity offers better accuracy than an LSM with *N*_*ens*_ number of liquids and higher percentage connectivity, we conducted an experiment. In other words, the goal of the experiment is to view the accuracy of two LSM configurations with same number of connections. The dominant component of the number of connections in an LSM is the connections between the excitatory neurons. Therefore, we varied the *P*_*E*→*E*_ for two LSM configurations (*N*_*ens*_ = 1 and *N*_*ens*_ = 4) and observed the accuracy for the TI46-alpha application. Figure 18 illustrates that for *P*_*E*→*E*_ < 0.57, the multiple liquid configuration (*N*_*ens*_ = 4) provides better accuracy, suggesting that the ensemble approach gives better results even under same number of connections in comparison to the single liquid baseline. For example, the ensemble approach gives ~83% accuracy at *P*_*E*→*E*_ = 0.4 and for the same number of connections, (i.e., at *P*_*E*→*E*_ = 0.1), the single liquid LSM configuration gives lower accuracy (~76%). However, for *P*_*E*→*E*_ > 0.57, single liquid LSM seems to perform better. Hence we conclude that at higher degrees of sparsity, the ensemble approach performs better than a single liquid baseline with the same number of connections.

Apart from the percentage connectivity, different connectivity patterns within the liquid were also considered in literature. For example, a probabilistic local connectivity within the liquid, inspired by the connectivity in biological neurons is suggested in Maass et al. (2003). We conducted an experiment with different sets of parameters (refer to the Supplementary Material) for the probabilistic local connectivity model. Our results indicate that, the highest accuracy achieved (for the ranges of parameters we have considered) with the probabilistic local connectivity model (an LSM with 1, 008 neurons arranged in a liquid column 6 × 6 × 28, gave a maximum accuracy of ~78%, for the TI-alpha speech recognition application) is lower than that attainable from our proposed ensemble approach (4 ensembles with 250 neurons in each, resulted in an accuracy of 83%, for the same TI-alpha application). More information on our analysis is included in the Supplementary Material.

### 3.5. Limitations of the Ensemble Approach

**Figure 19:**
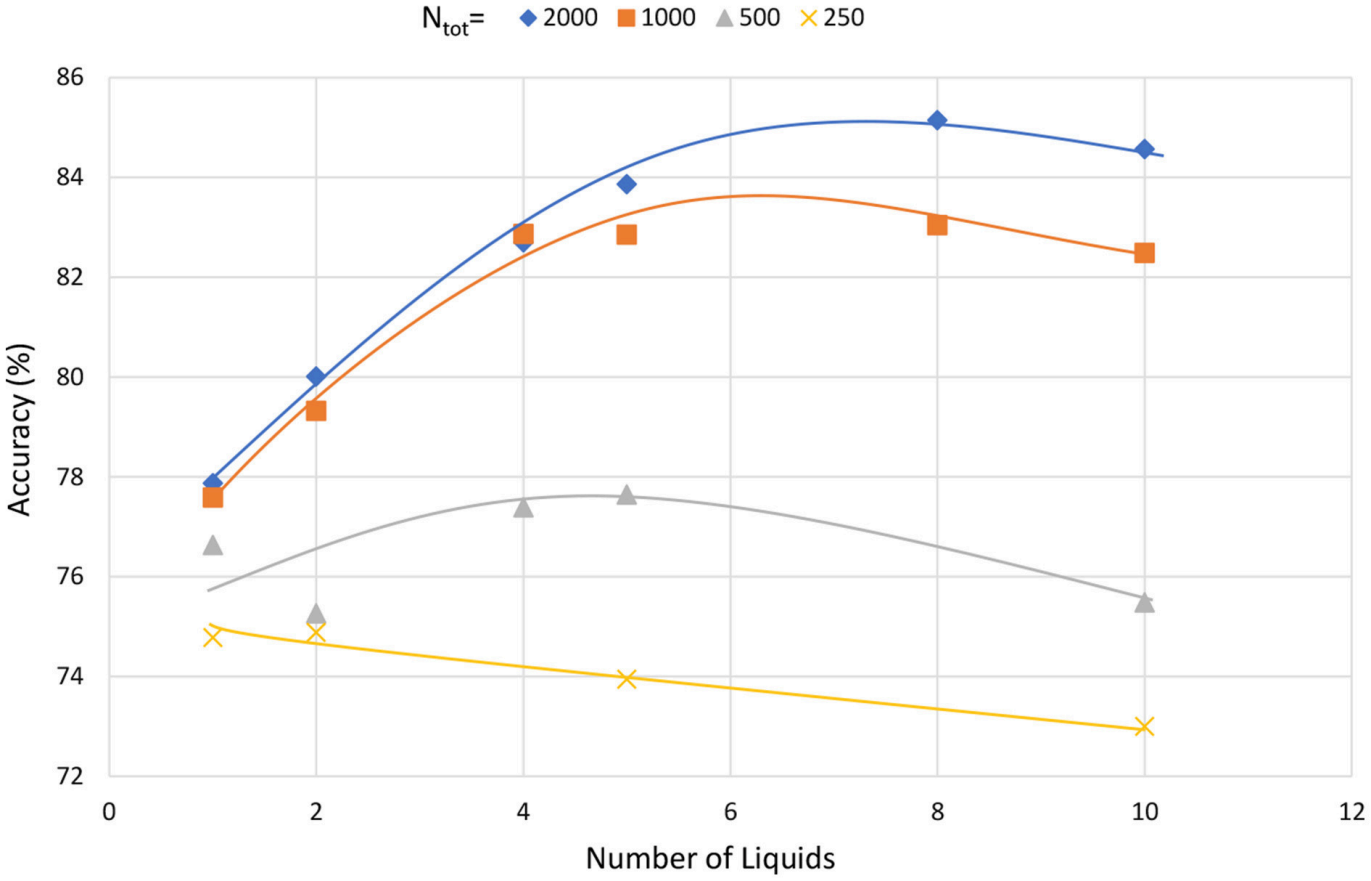
The accuracy varying with the number of liquids in the ensemble approach, for different total number of neurons (*N*_*tot*_). The LSM classifies speech data in the TI-alpha dataset.

In this section, we analyze whether dividing a liquid with any number of neurons (*N*_*tot*_) would result in similar accuracy improvements. To this effect, we created ensembles of liquids with different total number of neurons (*N*_*tot*_). As Figure 19 illustrates, liquids with large number of neurons show clear sign of accuracy improvement when divided into smaller liquids. However, when the number of neurons is smaller, dividing the liquid may result in decreased accuracy. For example, note that the accuracy reduces continuously when a liquid with 250 neurons is divided. This result is similar to the observation in Srinivasan et al. (2018), where the authors have shown that the input and liquid subdivision is beneficial for LSMs with large number of neurons. Similarly, here the ensemble approach makes sense only for LSMs with large number of neurons in them. In conclusion, we state the following with respect to the applicability of the ensemble approach for LSMs. In order to improve the accuracy of an LSM, the number of neurons can be increased. However, beyond a certain point, accuracy does not improve further. In such a case, the ensemble approach can be utilized to further increase the accuracy. Such accuracy improvements are not attainable by means of other simple methods that preserve the structural and training simplicity of the standard LSM, such as changing the connectivity.

### 3.6. Multiple Liquid-Multiple Readouts (MLMR) Approach

**Figure 20:**
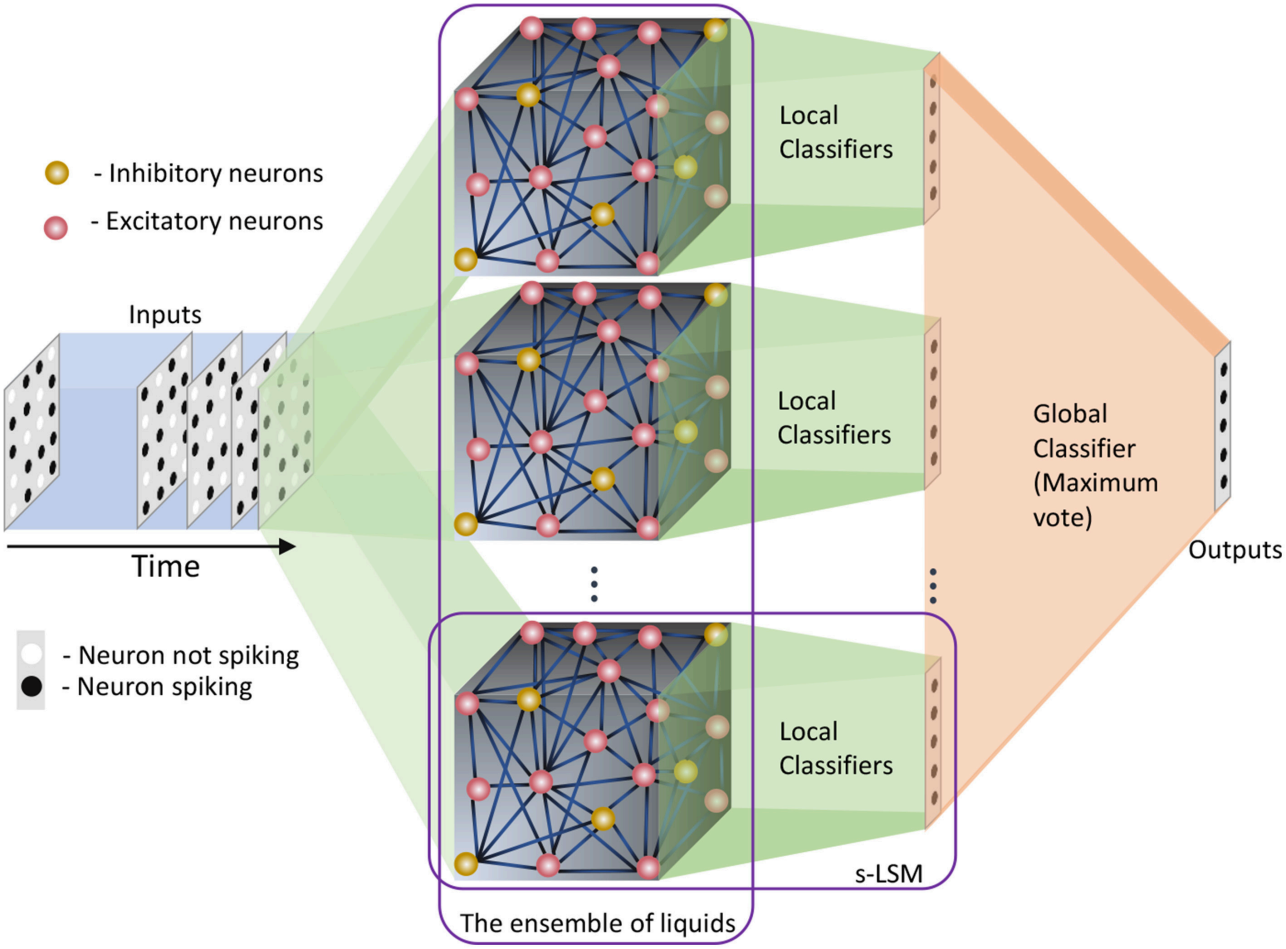
The structure of the multiple liquid-multiple readout (MLMR) approach. There are multiple small liquids, and individual classifiers at the end of each liquid. These are defined as small LSMs or s-LSMs. Final outcome (global output) is calculated by considering the maximum vote among all the local classified outputs from the s-LSMs (local outputs).

When moving from the single liquid approach to the ensemble of liquids approach, any benefit in terms of classifier training time was not observed. This is due to the fact that the number of total liquid neurons is the same, and we are using a single classifier. In this section, we analyze, if including a readout at the end of each small liquid is beneficial than having a single readout for all the liquids. The basic structure of this multiple liquid-multiple readouts (MLMR) approach is shown in Figure 20.

In contrast to our previous approach, this structure could be viewed as a collection of small LSMs (s-LSMs). Each s-LSM is trained individually, and the final classification is done by considering either the maximum outcome, or the majority vote among all the local classifiers. During training, we do not use all the training data points for each local s-LSM classifier. Instead, we divide the training space among the ensemble of s-LSMs based on the following two criteria:

1. Random training space division (RD)
2. Clustered training space division (CD)

**Figure 21:**
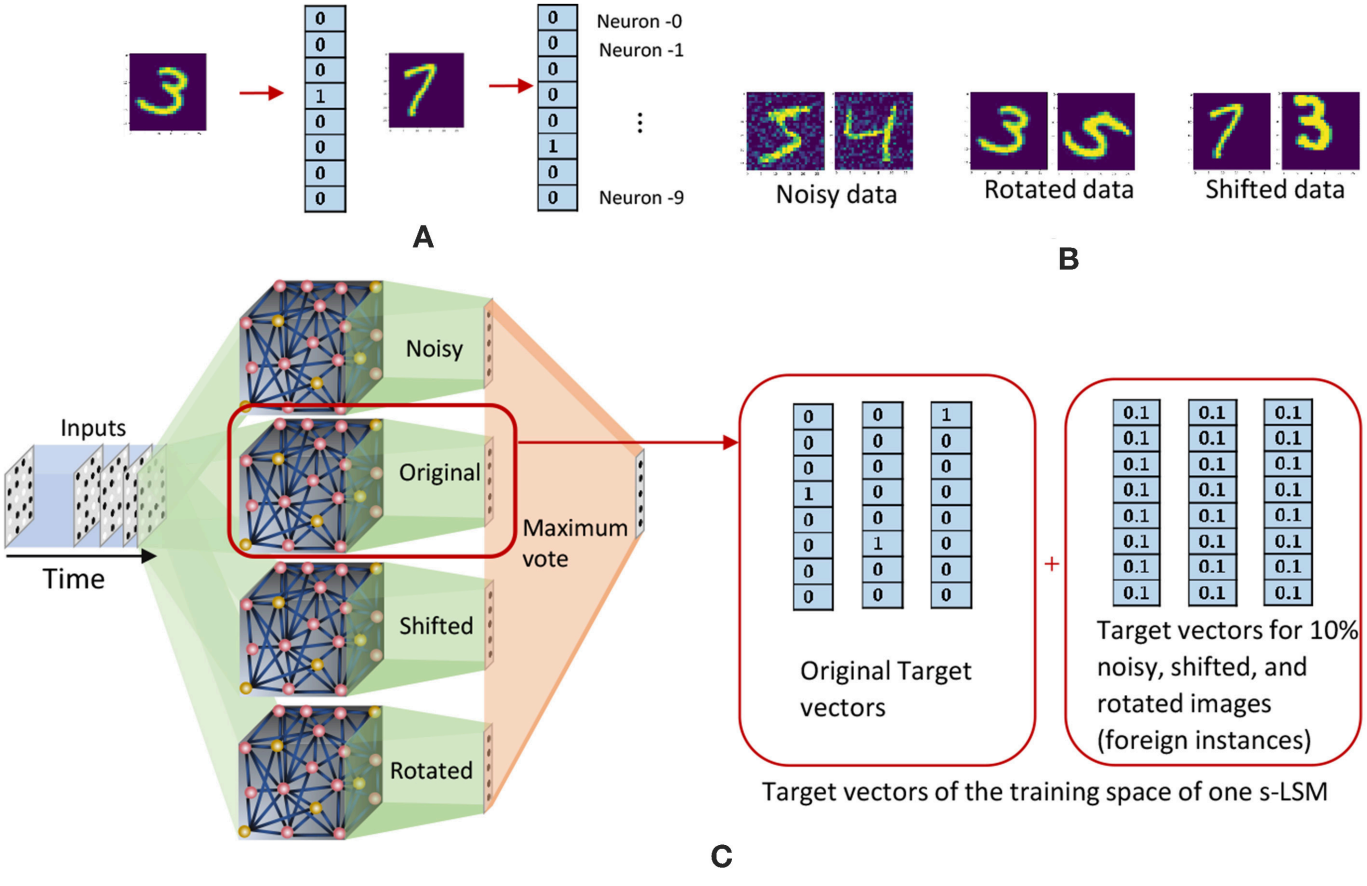
**(A)** Target vectors that correspond to images in the extended - MNIST data set. **(B)** Examples from the clustered training data space of the extended MNIST dataset. **(C)** The clustered training space division method. Each s-LSM is trained with a particular cluster of images, and an additional 10% of the images in the other clusters (foreign data). The target vectors of the foreign data are modified to have each value equal to 0.1.

In random training space division (RD) method, we randomly divide the training data space among the ensemble of s-LSMs, and feed them to obtain the corresponding liquid state vectors at the output of each liquid. These state vectors were then used to train the local classifiers attached to each s-LSM in the ensemble using gradient descent error backpropagation. For example, if there are *N*_*ens*_ number of s-LSMs and *N*_*train*_ number of examples in the training set, each s-LSM will be trained with $\frac{N_{train}}{N_{ens}}$ number of randomly picked training examples. On the other hand, in the clustered input space division (CD) method, we divide the training instances into certain clusters depending upon their features, (for instance, we have selected “original,” “rotated,” “shifted,” and “noisy” images from the extended MNIST dataset as clusters) and used them to train each readout. Here, an s-LSM has specific knowledge about the cluster of examples that it is trained with, and zero knowledge about other clusters. Therefore, an s-LSM may not correctly identify an input that belongs to a different cluster, apart from what it was trained with, leading to large accuracy degradation at the global classifier. For example, if a rotated image of digit “1” is given as the input, the s-LSM that was trained with rotated images will correctly recognize the given image. i.e., the output neuron−1 gives the highest outcome (there are 10 output neurons and they are indexed as neuron−0 through neuron−9 as shown in Figure 21). Other s-LSMs may not recognize this input correctly, potentially leading to another neuron apart from neuron−1 to give a high value at the outputs of their corresponding classifiers. When getting the final outcome using “maximum output” method, the neuron that gives the highest value over all the s-LSMs may not be neuron−1. Instead, it could be some different neuron from an s-LSM that was not trained with rotated images. To address this issue, we use an “inhibition” criterion to suppress the s-LSMs from giving high outputs for cluster types that they are not trained with. Initially we divide the training space into clusters along with their standard target vectors (vectors of which the length is equal to the number of classes *L*. If the input belongs to the *i*th class, then the *i*th element in the vector will be “1” and the other elements will be “0.” Refer to Figure 21). Then, we randomly select 10% of the training instances from each cluster (foreign instances), and add them to the training space of all other clusters. The target vectors of the foreign instances are forced to have all their elements equal to 1/*L* (*L* is the number of classes) and we name this target vector as “inhibitory label vector”. This will force each s-LSM outcome to be low, when the presented input does not belong to the cluster with which the s-LSM was trained. This method is explained graphically in Figure 21, by means of an example.

We used the handwritten digit recognition application with the extended MNIST data set, to check the accuracy, performance, and training time of the aforementioned methods. The training data set was divided into 4 clusters; original MNIST images, noisy images, rotated images and shifted images. Total number of neurons were 1, 000 and each s-LSM has 250 neurons. The connectivity is set as indicated in Table 1. Table 3 reports the accuracy of the above explained two training space division methods (RD and CD) along with the accuracy of the baseline (single liquid with 1, 000 neurons). The accuracy of the two methods (RD → 82.5% and CD → 83.1%) are inferior to that of the baseline (86.9%).

**Table 3:** Accuracy of different ensemble approaches.

**Approach (total number of neurons= **1, 000**)**	**Accuracy (%)**
Single liquid, single readout (baseline)	86.9
4 ensembles, single readout (MLSR)	89.0
4 ensembles, 4 readouts, (MLMR) random training space division (RD)	82.5
4 ensembles, 4 readouts, (MLMR) clustered training space division (CD)	83.1

**Figure 22:**
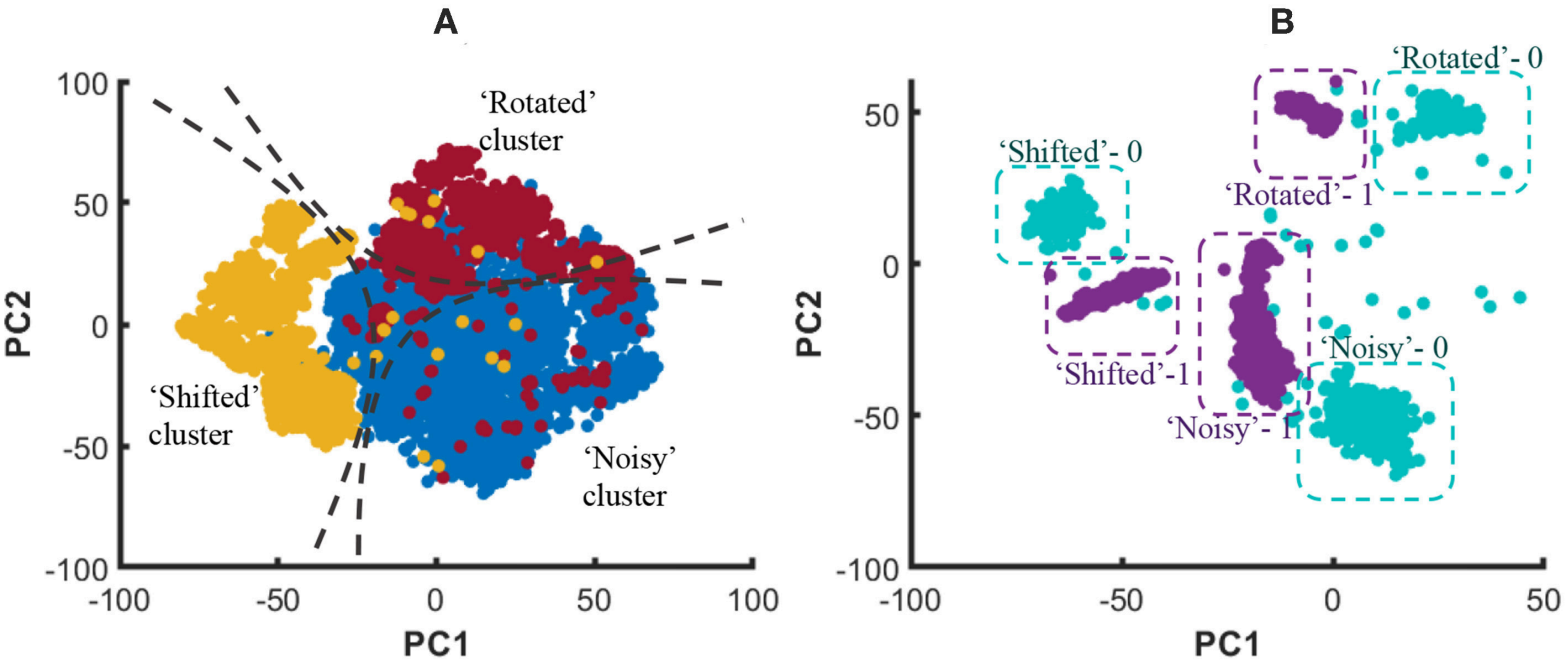
**(A)** The t-Distributed Stochastic Neighbor Embedding (t-SNE) of the high dimensional input data points, in 2D space for better visualization. The distribution of data points that belong to three clusters (“Noisy,” “Shifted,” and “Rotated”) stay spatially separated. **(B)** The distribution of data points of digit “1” and digit “0” that belongs to three clusters.

When comparing with RD method, CD gives better accuracy for the same number of neurons. The reason for this can be explained as follows. The clusters in the training dataset can have different overlapping/non-overlapping distributions. For instance, three clusters (“noisy,” “shifted,” and “rotated”) in the considered example in this work follow three different distributions as shown in Figure 22A. The figure elaborates the t-Distributed Stochastic Neighbor Embedding (t-SNE) (Maaten and Hinton, 2008) of the high dimensional images that belong to the aforementioned three clusters, for better visualization in the lower dimensional space (2D). Due to this separate distributions, examples that belong to the same class but in different clusters may not spatially stay together in the higher dimensional space. For example, Figure 22B shows the data points that correspond to digit “0” and digit “1” in different clusters, and neither the data points of digit “0” nor “1” stay together. Let us consider the RD method, and how it tries to classify the aforementioned digit “0” and digit “1.” If there are *N*_*tot*_ amount of training examples and *L* classes, the number of examples that belongs to class *i* each classifier sees is $N_{ex,i}=\frac{N_{tot}}{L\times N_{ens}}$. The *N*_*ex, i*_ number of examples a classifier in RD method sees belongs to *N*_*ens*_ number of clusters and they are distributed all over as shown in Figure 22B. According to the figure, the two classes are not linearly separable. Therefore, the RD method leads to more misclassifications as elaborated in Figure 22A. In contrast, a classifier trained for “shifted” data cluster in CD method fits to a decision boundary that classifies digit “0” and digit “1” that *onlybelongs* to “shifted” data cluster. Owing to the proposed inhibition criterion, the classifier trained for “shifted” examples in CD tries to put the data points that belong to other clusters into a single category. As the Figure 22B illustrates, the classes: “digit 0,” “digit 1,” and “foreign” are more linearly separable by CD method than the RD method, and this leads to higher accuracy in RD method.

**Figure 23:**
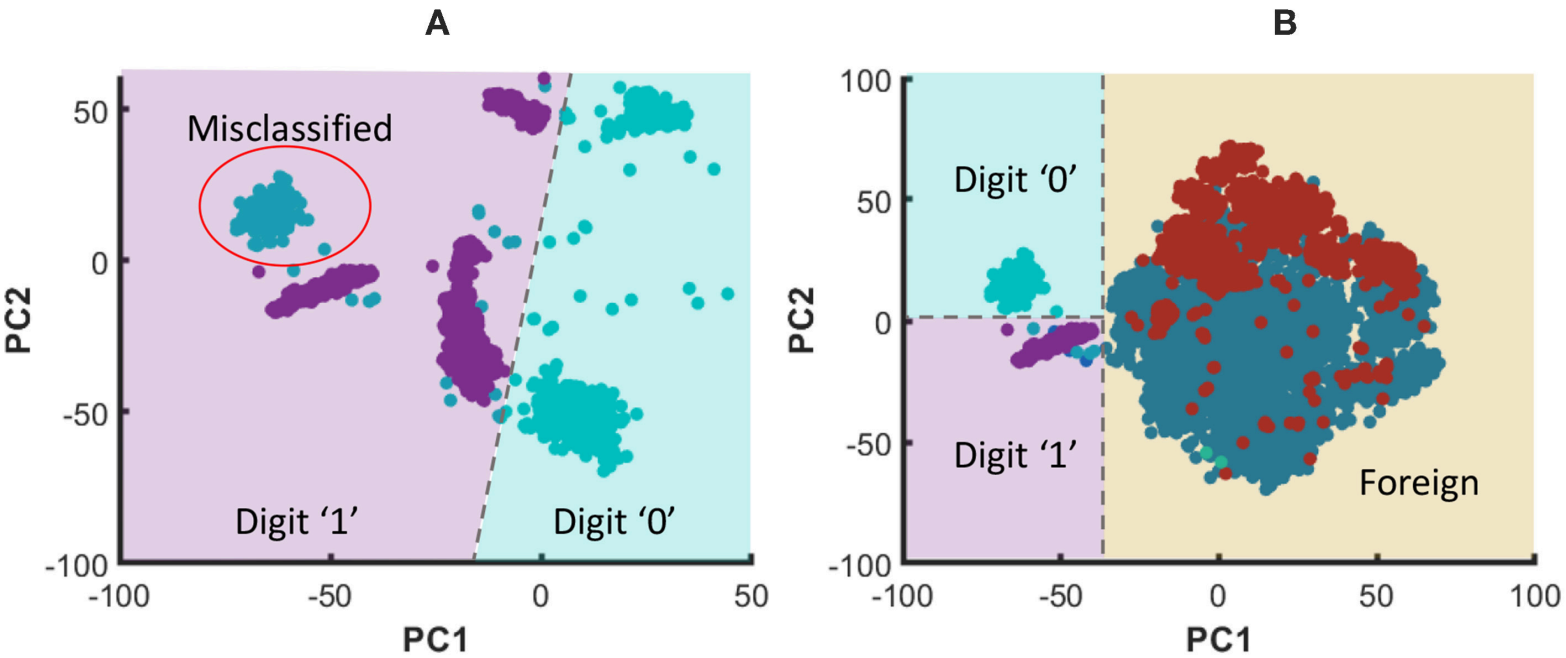
**(A)** The graphical representation of the RD method trying to classify digit “0” and digit “1” that belong to three clusters using a linear classifier. Note that the digit “0” that belong to the shifted cluster is misclassified as digit “1”. **(B)** The graphical representation of the CD method trying to classify digit “0” and digit “1”. The particular classifier shown has learned to correctly classify digit “0” and digit “1” that belong to “shifted” cluster. Furthermore, it recognizes the data points that belong to foreign clusters due to the proposed inhibition criterion. The dashed lines show the classifier decision boundaries.

Selecting more foreign examples would result in the classifier to concentrate more on fitting the foreign data into “nhibitory label vectors,” instead of classifying data in the corresponding cluster. Consider adding an *x*_*f*_% of foreign instances per cluster. This would result in adding $\frac{\left(N_{ens}-1\right)x_{f}\%}{\left(N_{ens}-1\right)x_{f}\%+1}$ overall percentage of extra data that does not belong to the cluster to which the classifier must be trained. The training space of the classifier that must be trained for “shifted” images consists of the following “sets”: (1) shifted digit “0,” (2) shifted digit “1,”…, 10)shifted digit “9,” randomly selected images from (11) “noisy” cluster, (12) “original” cluster, (13) “rotated” cluster. We selected a percentage, that will pick approximately equal number of data points from each of the aforementioned 13 sets. In our particular example, to make ~7.7%(= 100/13) of the training space to be attached to each of the above “sets”, *x*_*f*_% needs to be selected as 10%. The total percentage of the foreign instances are 23% ($=\frac{\left(N_{ens}-1\right)x_{f}\%}{\left(N_{ens}-1\right)x_{f}\%+1}$) per cluster.

**Figure 24:**
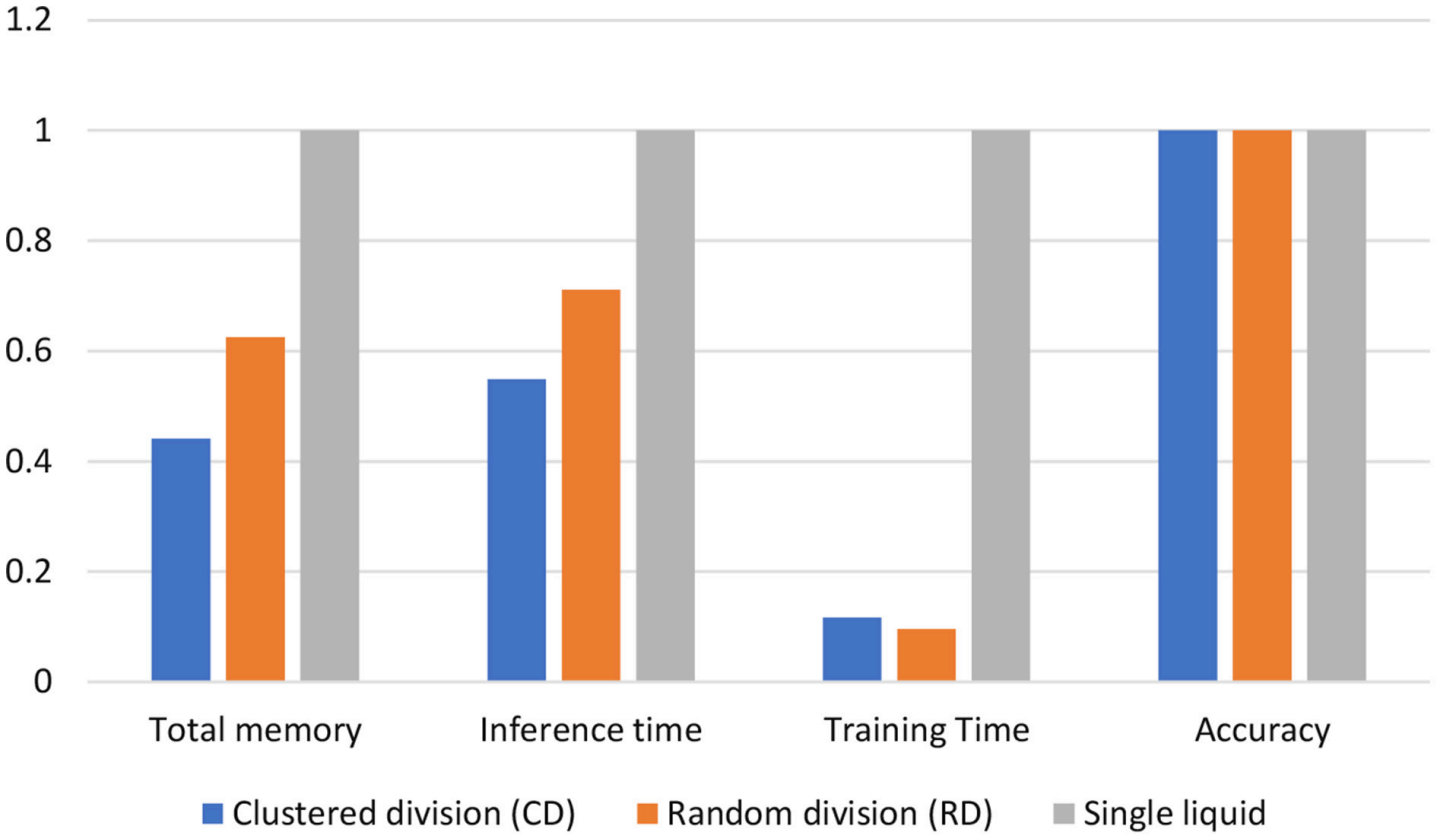
Normalized total memory requirement, inference time, and training time of the clustered training space division method (CD), random training space division method (RD), and the single liquid baseline. The results are under iso-accuracy conditions.

In order to see if there is any benefit in the MLMR approach when achieving a “given” accuracy, we reduced *N*_*tot*_ in the baseline to match the accuracy of both the RD and CD methods. The memory requirement, inference time, and training time were calculated for two scenarios. First, *N*_*tot*_ in the baseline was selected such that both the baseline and the RD method have the same accuracy (82.5%). Second, *N*_*tot*_ in the baseline was selected such that it matches the accuracy of the CD method (83.1%). In each of the above scenarios, the obtained memory requirement, inference time, and training time values were normalized with respect to the baseline. These normalized values for the two cases are shown in a single graph in Figure 24. The CD method is better in terms of memory requirement and inference time, in comparison to the single liquid baseline and RD method. We calculated the total number of MAC (multiply and accumulate) operations during training to estimate the training time (it is a function of the number of neurons in a liquid, number of output neurons, and number of training examples). Lowest training time was achieved in the RD method. The CD method offers 56% reduction in memory and 45% reduction in inference time, with respect to the baseline. For a 1, 000 total number of neurons, the 4 ensemble case with a single classifier (studied in section 3.2. Let us denote this method as multiple liquids, single readout or MLSR approach) resulted in 78% reduction in memory usage and 72% reduction in inference time along with 2.1% accuracy improvement (hence better than both CD and RD methods under the memory usage and inference time metrics). However, in terms of training time, the MLSR approach did not show any improvement, whereas the MLMR showed 88% reduction with respect to the baseline.

## 4. Conclusion

We have presented an ensemble approach for Liquid State Machines (LSMs) that enhances separation and approximation properties, leading to accuracy improvements. The separation property in LSMs measures the dispersion between projected liquid states from different classes, whereas the approximation property indicates the concentration of the liquid states that belong to the same class. The ratio between SP and AP (*DR*) is a measure of the class discrimination. We witnessed that the *DR* increases when a large liquid is divided into multiple smaller independent liquids across four speech and image recognition tasks. We observed the existence of an optimal number of liquids (*N*_*ens, opt*_) until which the *DR* increases and saturates thereafter. Owing to the improvement in the *DR* in our proposed ensemble approach, we noticed an LSM accuracy enhancement with increasing number of liquids. The accuracy peaked at the same *N*_*ens, opt*_ point at which each *DR* saturated, for different recognition tasks. This validated the existence of an optimal number of liquids which gives the best accuracy for the LSM, and this point is highly dependent upon the application and the total number of liquid neurons.

There is plethora of complex approaches that concentrate on improving the accuracy of LSMs, including learning the liquid connections (Wang and Li, 2016; Xue et al., 2016). In contrast to such works, our proposed approach does not change the simple structure and training methods of LSMs. Furthermore, the ensemble approach gives better accuracy when compared with other simple mechanisms of improving the LSM accuracy such as increasing the number of neurons, changing the percentage connectivity, and utilizing the probabilistic local connectivity models. Apart from providing improved accuracy, the proposed ensemble approach comes with other benefits including lower memory requirement and lower inference time. We have shown that creating an ensemble of liquids leads to lower inter-connections in comparison to a single liquid with the same number of neurons. Furthermore, the liquid evaluation can potentially be parallelized in the ensemble approach due to the existence of small independent liquids. This results in reduced LSM inference time. The accuracy improvement with increasing number of liquids in the ensemble becomes less evident when the total number of neurons is small. In fact, creating an ensemble of liquids with a small number of neurons will rather reduce the accuracy. Hence the ensemble approach makes sense for LSMs with large number of neurons (Srinivasan et al., 2018).

Since there is no benefit in terms of training time between a single-liquid LSM and the proposed ensemble approach (MLSR), we investigated the MLMR approach where a classifier is added to each small liquid in the ensemble. By dividing the training example space to train each small LSM, we were able to attain significant benefits in terms of training time, when compared with MLSR approach. There are multiple classifiers that were trained independently in the MLMR approach, and the final output is the maximum vote of all the local classifiers. The set of multiple liquid-classifier units are in fact a collection of small LSMs (noted as s-LSMs). Despite the performance benefits during training, we noticed an accuracy degradation in the MLMR approach, when compared with both the MLSR approach and the single-liquid baseline LSM with equal number of liquid neurons. The reason for this can be explained as follows. The classifiers in each s-LSM are smaller than that of the baseline and the MLSR approaches. A large classifier (as in the baseline and MLSR approach) has more number of parameters and is capable of fitting in to an unknown function better than a small classifier (Krzywinski and Altman, 2015), leading to improved accuracy.

## Data Availability

The datasets generated for this study are available on request to the corresponding author.

## Author Contributions

PW performed the simulations. All the authors contributed in developing the concepts, generating experiments, and writing the manuscript.

### Conflict of Interest Statement

The authors declare that the research was conducted in the absence of any commercial or financial relationships that could be construed as a potential conflict of interest.
