# Supplementary material for: Analysis of Liquid Ensembles for Enhancing the Performance and Accuracy of Liquid State Machines
Source: Front Neurosci. 2019 May 28;13:504. doi: 10.3389/fnins.2019.00504 (PMC6546930; doi:10.3389/fnins.2019.00504)
Supplement: Supplementary file 1 [file Data_Sheet_1.PDF]

Supplementary material for ‘Analysis of Liquid Ensembles for Enhancing the Performance and Accuracy of Liquid State Machines’

Parami Wijesinghe [1,*], Gopalakrishnan Srinivasan [1], Priyadarshini Panda [1] and Kaushik Roy [1]

[1] *Purdue University, School of Electrical and Computer Engineering, West Lafayette, Indiana, 47907 USA*

Correspondence*:
Parami Wijesinghe
pwijesin@purdue.edu

1 PROBABILISTIC LOCAL CONNECTIVITY WITHIN THE LIQUID

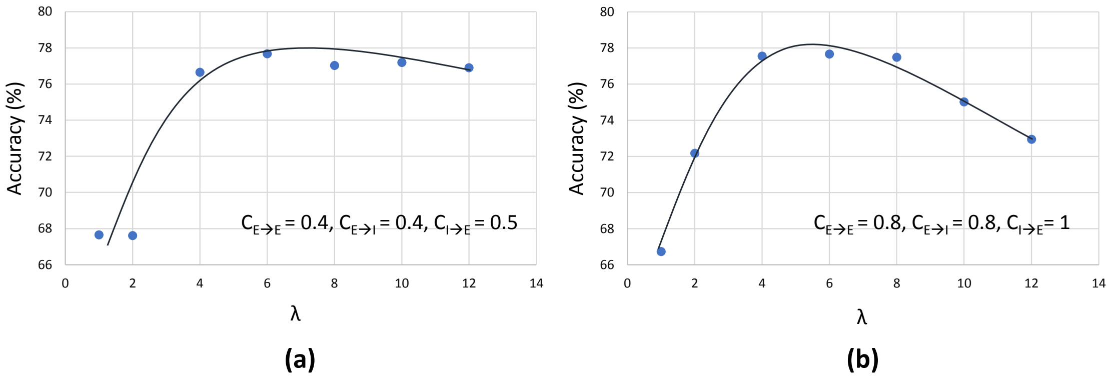

Figure 1. The accuracy varying with λ, for two different sets of C parameter selections, (a) $C_{E\to E} = 0.4, C_{E\to I} = 0.4, C_{I\to E} = 0.5$, and (b) $C_{E\to E} = 0.8, C_{E\to I} = 0.8, C_{I\to E} = 1.0$. This is a single liquid-LSM, which classifies speech patterns in the TI-alpha dataset. There are 1008 neurons in the liquid which are arranged in a $6 \times 6 \times 28$ sized 3D column.

In order to improve the accuracy of an LSM, different connectivity patterns within the liquid were considered in literature. For example, the work in Maass et al. (2003) suggests local connectivity within the liquid, inspired by the connectivity in biological neurons. It has been shown that in the mammalian cortex, the neurons are typically connected to its neighbors only (Markram et al., 1998). As explained in Maass et al. (2002b), Markram et al. (1998), and Maass et al. (2002a), the further apart two neurons are, more ‘likely’ it is that they are not connected to each other. The existence of a connection between two neurons (all the neurons were arranged in a 3D lattice initially) is hence modeled as a probabilistic function (P_{con}) of the Euclidean distance ($D^2(a, b)$) between neuron a and b. λ is a parameter which controls both the average number of connections and the average distance between two neurons. C is the highest probability that two neurons are connected (occurs at $\lambda \to \infty$). Multiple values of C ($i.e.$, $C_{E\to E}, C_{E\to I}, C_{I\to E}$) can

be used depending upon the type of pre- and post-neurons being connected. The probabilistic function P_{con} is as described by the following equation:

$$P_{con} = Ce^{-\frac{D^2(a,b)}{\lambda^2}} \tag{1}$$

Figure 1 shows how the accuracy varies with λ for two different sets of C values in a liquid that has 1008 neurons. The application is recognizing the utterances of the letters in the English alphabet, in the TI46 speech corpus (TI-alpha). The neurons were arranged in a 3D column ($6 \times 6 \times 28$) approximately preserving the dimensional ratios of the neural microcircuit column proposed in Maass et al. (2003). As Figure 1 illustrates, the highest accuracy attained was $\sim 78\%$. The accuracy of an LSM (1000 neurons, refined $In - E$, $E - E$, $I - E$ and $E - I$ percentage connectivity) with randomly pruned connections (without considering the probabilistic local connectivity model) was 77.6% accuracy for the same application.

The ensemble approach, to a certain extent, is similar to the aforementioned notion of 'local connectivity', since we split up a large liquid into multiple smaller liquids which avoids long connections. In the probabilistic local connectivity model, the dynamics of each neuron in the liquid are dependent upon that of other neurons, since they are all directly or indirectly connected. In contrast, the ensemble approach allows multiple small networks to be evaluated in parallel since they are independent, making the evaluation time smaller. Furthermore, the accuracy of four liquids with 250 neurons in each is 83%, which is considerably higher than the highest accuracy attainable through probabilistic local connectivity (78% for 1008 neurons) model, for the same speech application.

REFERENCES

Maass, W., Legenstein, R., and Markram, H. (2002a). A new approach towards vision suggested by biologically realistic neural microcircuit models. In *International Workshop on Biologically Motivated Computer Vision* (Springer), 282–293

Maass, W., Natschläger, T., and Markram, H. (2002b). Real-time computing without stable states: A new framework for neural computation based on perturbations. *Neural computation* 14, 2531–2560

Maass, W., Natschläger, T., and Markram, H. (2003). A model for real-time computation in generic neural microcircuits. In *Advances in neural information processing systems*. 229–236

Markram, H., Wang, Y., and Tsodyks, M. (1998). Differential signaling via the same axon of neocortical pyramidal neurons. *Proceedings of the National Academy of Sciences* 95, 5323–5328
